# Type I IFN signaling blockade by a PASylated antagonist during chronic SIV infection suppresses specific inflammatory pathways but does not alter T cell activation or virus replication

**DOI:** 10.1371/journal.ppat.1007246

**Published:** 2018-08-24

**Authors:** Krystelle Nganou-Makamdop, James M. Billingsley, Zachary Yaffe, Gregory O’Connor, Gregory K. Tharp, Amy Ransier, Farida Laboune, Rodrigo Matus-Nicodemos, Andrea Lerner, Lavina Gharu, Jennifer M. Robertson, Mandy L. Ford, Martin Schlapschy, Nadine Kuhn, Alexandra Lensch, Jeffrey Lifson, Martha Nason, Arne Skerra, Gideon Schreiber, Steven E. Bosinger, Daniel C. Douek

**Affiliations:** 1 Human Immunology Section, Vaccine Research Center, National Institute of Allergy and Infectious Disease, National Institutes of Health, Bethesda, Maryland, United States of America; 2 Division of Microbiology and Immunology, Emory Vaccine Center, Yerkes National Primate Research Center, Atlanta, Georgia, United States of America; 3 Department of Surgery and Emory Transplant Center, Emory University School of Medicine and Emory Healthcare, Atlanta, GA; 4 Lehrstuhl für Biologische Chemie, Technische Universität München, Freising (Weihenstephan), Germany; 5 AIDS and Cancer Virus Program, Frederick National Laboratory for Cancer Research, Frederick, Maryland, United States of America; 6 Biostatistics Research Branch, Division of Clinical Research, National Institute of Allergy and Infectious Diseases, National Institutes of Health, Bethesda, Maryland, United States of America; 7 XL-protein GmbH, Freising, Germany; 8 Department of Biomolecular Sciences, Weizmann Institute of Science, Rehovot, Israel; 9 Department of Pathology & Laboratory Medicine, Emory University, Atlanta, Georgia, United States of America; University of Illinois at Chicago College of Medicine, UNITED STATES

## Abstract

Chronic activation of the immune system in HIV infection is one of the strongest predictors of morbidity and mortality. As such, approaches that reduce immune activation have received considerable interest. Previously, we demonstrated that administration of a type I interferon receptor antagonist (IFN-1ant) during acute SIV infection of rhesus macaques results in increased virus replication and accelerated disease progression. Here, we administered a long half-life PASylated IFN-1ant to ART-treated and ART-naïve macaques during chronic SIV infection and measured expression of interferon stimulated genes (ISG) by RNA sequencing, plasma viremia, plasma cytokines, T cell activation and exhaustion as well as cell-associated virus in CD4 T cell subsets sorted from peripheral blood and lymph nodes. Our study shows that IFN-1ant administration in both ART-suppressed and ART-untreated chronically SIV-infected animals successfully results in reduction of IFN-I-mediated inflammation as defined by reduced expression of ISGs but had no effect on plasma levels of IL-1β, IL-1ra, IL-6 and IL-8. Unlike in acute SIV infection, we observed no significant increase in plasma viremia up to 25 weeks after IFN-1ant administration or up to 15 weeks after ART interruption. Likewise, cell-associated virus measured by SIV gag DNA copies was similar between IFN-1ant and placebo groups. In addition, evaluation of T cell activation and exhaustion by surface expression of CD38, HLA-DR, Ki67, LAG-3, PD-1 and TIGIT, as well as transcriptome analysis showed no effect of IFN-I blockade. Thus, our data show that blocking IFN-I signaling during chronic SIV infection suppresses IFN-I-related inflammatory pathways without increasing virus replication, and thus may constitute a safe therapeutic intervention in chronic HIV infection.

## Author summary

Innate and adaptive immune activation is one of the strongest predictors of HIV disease progression to AIDS and non-AIDS morbidity and mortality. Type I interferon (IFN-I) signaling is a major driver of such activation even in ART-treated people. Therefore, manipulation of IFN-I signaling has been proposed as a therapeutic approach to improve HIV disease outcome. However, we previously reported that blockade of IFN-I signaling in rhesus macaques during acute SIV infection results in increased SIV reservoir size, accelerated CD4 T cell depletion and faster progression to AIDS. Our present study addresses the more clinically relevant effects of IFN-I signaling blockade in chronic SIV infection during treatment with antiretroviral therapy. We found that administration of a novel, long half-life, PASylated IFN-I receptor antagonist suppressed IFN-I-related inflammatory pathways but, in contrast to the case of acute SIV infection, did not result in loss of control of SIV replication. Thus, our findings provide a rationale for the safe and effective use of such interventions to reduce inflammation in HIV-infected people during ART.

## Introduction

Persistent inflammation during chronic HIV infection is a central contributing factor to immune exhaustion, CD4 T cell depletion and progression to AIDS [1–3]. Previous studies aimed at understanding the nature of this immune dysfunction have revealed a key role for type I interferons (IFN-I). IFN-I has been shown to suppress HIV infection in vitro [4] and SIV infection in rhesus macaques in vivo [5]. Studies in non-human primates have demonstrated a link between type I IFN responses and pathogenic SIV infection [6–8]. While IFN-I signaling resulting from SIV infection waned during the transition from acute to chronic non-pathogenic infection in SIV natural hosts African green monkeys and sooty mangabeys, a persistent response was associated with pathogenic infection and progression to AIDS in experimental SIV infection of rhesus and pigtail macaques. In humans, plasma levels of IFN-I have been shown to correlate directly with plasma HIV RNA and inversely with CD4 T cell count [9]. Moreover, administration of IFN-I to HIV-infected persons resulted in lower CD4 T cell counts [10] and increased CD8 T cell activation [11]. These findings attributed, at least in part, the severity of infection and exacerbation of the disease to type I IFN signaling and raised considerable interest in the potential therapeutic benefits of blocking IFN-I during infection. Associations between IFN-I and chronic viral infections have led to numerous studies where IFN-I signaling was manipulated [12]. Blockade of IFN-I signaling with anti-type I IFN receptor (IFNAR) antibody in murine LCMV infection resulted in reduced immune activation and improved viral clearance [13, 14]. Recently, two independent studies showed that administration of anti-IFNAR antibodies to ART-suppressed, HIV-infected humanized mice resulted in reduced immune activation and lowered reservoir size [15, 16]. The efficacy of IFN-blockade in the mouse models has provided a rationale for testing in the SIV-infected non-human primates. In our prior study, we found that blocking IFN-I during acute SIV infection in rhesus macaques resulted in reduced expression of antiviral genes, increased size of the SIV reservoir and accelerated CD4 T cell depletion and progression to AIDS [5]. Quite reasonably, this adverse outcome of IFN-I blockade during acute infection raised major concerns for the safety of IFN-I blockade during chronic HIV infection. As treatment with antiviral drugs reduces but does not completely normalize inflammation and IFN-I signaling [2, 17, 18], it is important to assess the effects of manipulation of IFN-signaling during chronic infection under ART. Thus, our primary objective in the present study was to test the effect of IFN-blockade on both inflammatory status and the control of viral replication during ART-treated and untreated chronic SIV infection in monkeys, and thus to establish the safety profile of this experimental therapy for clinical use in ART-treated HIV infection.

## Results

### ART and PASylated IFN-I antagonist administration

**Fig 1:**
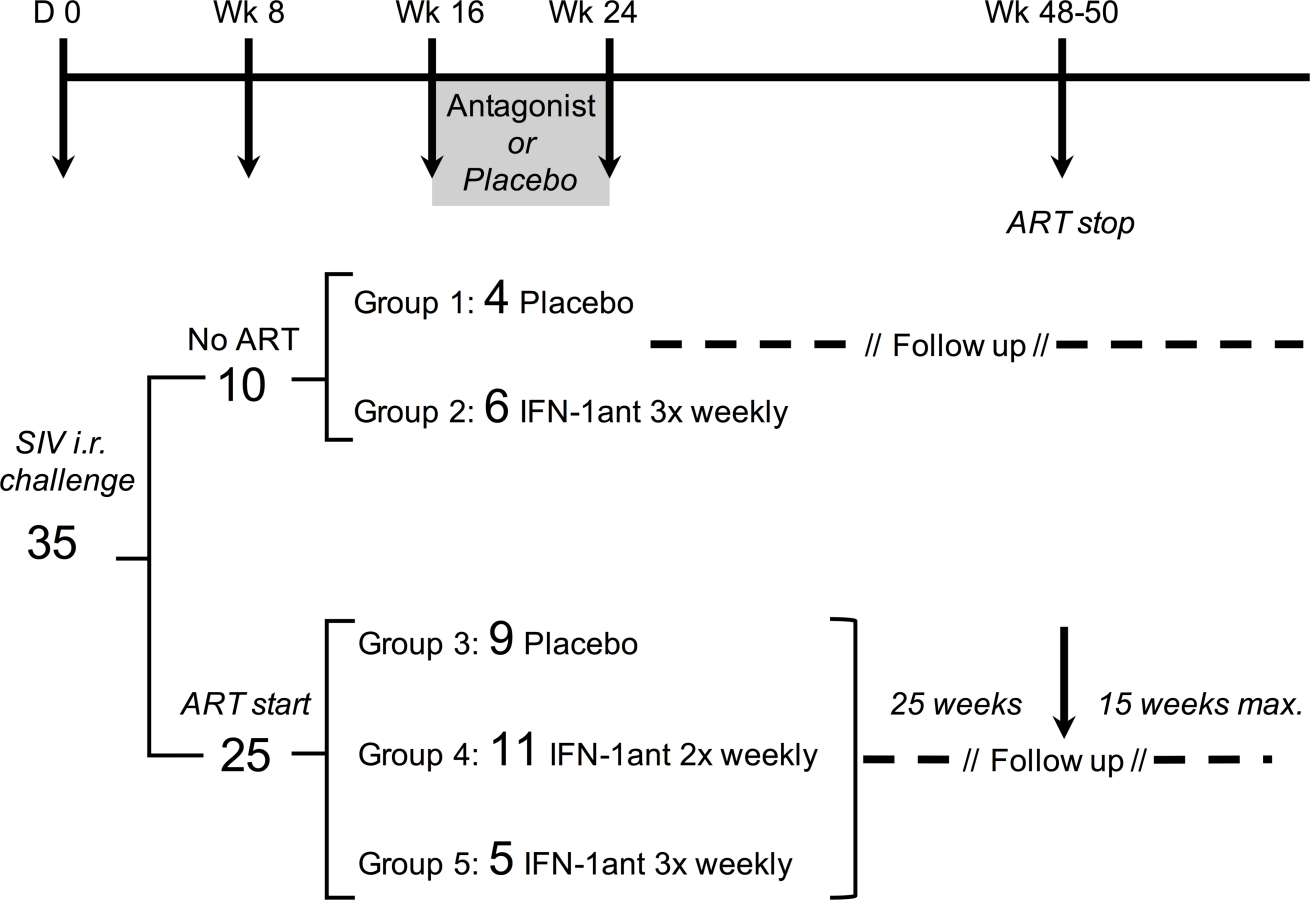
Experimental design. 35 rhesus macaques received intrarectal challenge with SIV_MAC251_ at baseline. At week 8 post-infection (p.i.), 25 animals received ART and 10 animals remained untreated. From week 16 to week 24 p.i., ART untreated SIV-infected animals received placebo saline (group 1, n = 4) and PASylated IFN-1ant 3 times per week (group 2, n = 6). ART-treated SIV-infected macaques received placebo saline (group 3, n = 9), PASylated IFN-1ant 2 times per week (group 4, n = 11) or 3 times per week (group 5, n = 5) from week 16 to week 24 p.i.. About 25 weeks after IFN-1ant, ART was interrupted and all animals monitored for an additional 15 weeks.

Eight weeks after rectal SIV_MAC251_ challenge, 25 rhesus macaques initiated daily ART while 10 remained untreated (Fig 1). To block type I IFN signaling, infected animals were treated with a PASylated type I interferon receptor antagonist (IFN-1ant) [5] which was obtained by fusion of the native IFN-1 antagonist with a 600 residue conformationally disordered chain of Pro/Ala/Ser amino acids [19], leading to a significantly prolonged plasma half-life of 19.4 ± 2.6 h in rhesus macaques and a 70-fold enhanced area under the curve compared with the unmodified IFN-1 antagonist (S1 Fig), while maintaining high receptor binding activity with K_D_ = 451 ± 3 pM. Antagonist or saline placebo were administered either twice or three times per week from weeks 16 to 24 post-infection, with 3x weekly doses resulting in higher plasma concentrations compared to 2x weekly injections. Among the animals that did not receive ART, 4 placebo (group 1) and 6 IFN-1ant-treated animals (group 2) received 3x weekly injections from week 16 to week 24 and had no significant difference in plasma virus load (VL) before administration of the antagonist (S2 Fig). In the ART-treated groups, 9 placebo-treated animals (group 4) and 11 IFN-1ant-treated animals (group 4) received twice weekly injections while a set of 5 animals with a higher initial median plasma virus load (group 5) received 3 times weekly IFN-1ant. Despite significantly higher plasma VL from week 4 to 5 post-infection in the 3 times weekly IFN-1ant group, Plasma VL at start of ART (week 8) and at start of antagonist administration (week 16) showed no statistical difference between the groups.

### ISG expression and efficacy of IFN-I blockade

**Fig 2:**
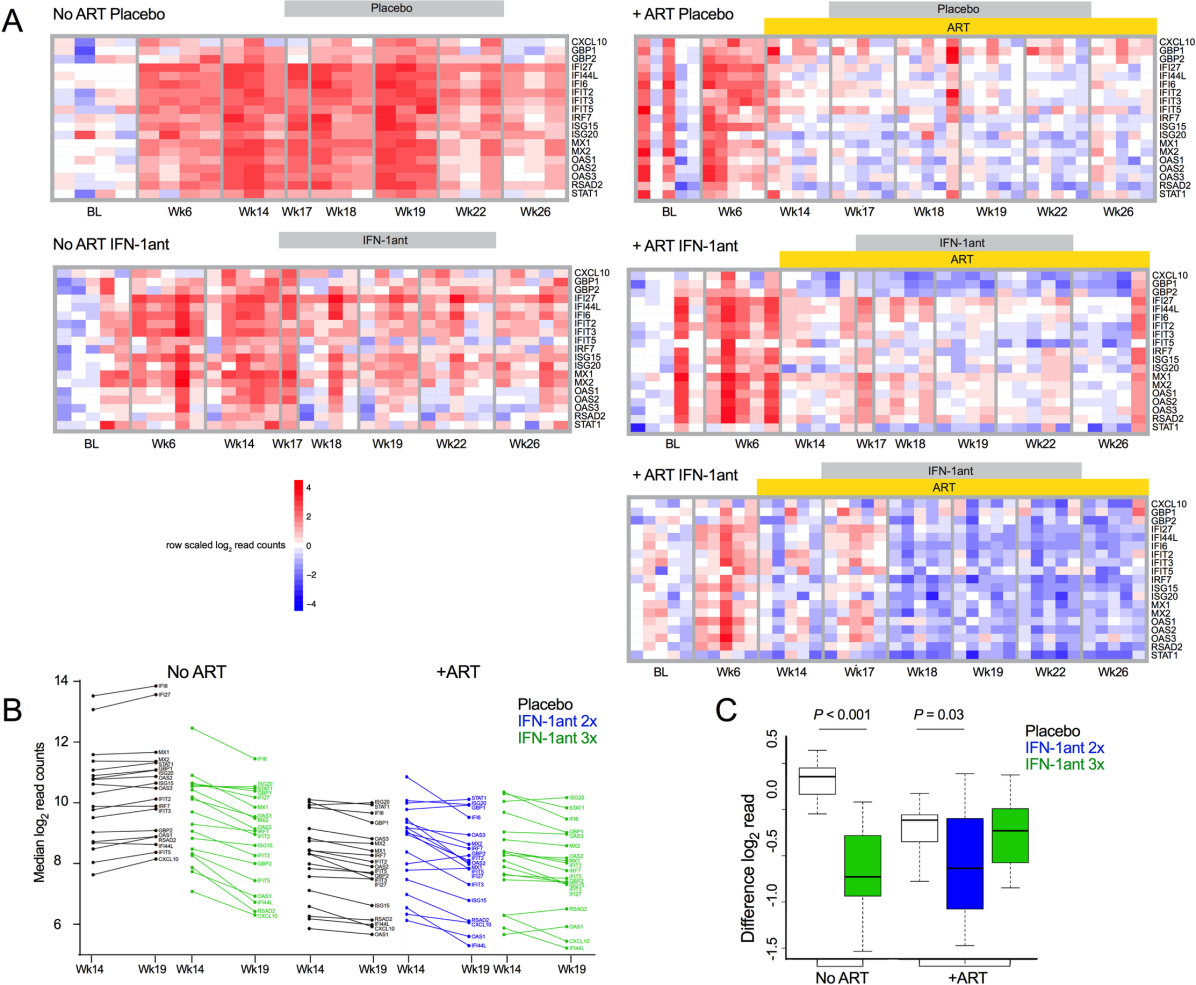
Effect of PASylated IFN-1ant on ISG expression levels. Expression of ISGs assessed by RNA sequencing in ART-treated and ART-untreated macaques from SIV challenge at baseline throughout administration of PASylated IFN-1ant (3x weekly or 2x weekly) or placebo saline from weeks 16 to 24 p.i. (**a**) Heatmap colors represent log_2_ transformed library size normalized read counts scaled to unit variance across transcript vectors (rows), and normalized to the baseline median sample value of each transcript. Line plots (**b**) represent the median log_2_ transformed gene expression estimates for each ISG at week 14 and week 19. Box-whisker plots (**c**) summarize the differences between weeks 14 and 19 median gene expression estimates for each gene. Error bars indicate lower and upper quartiles. *P* values were calculated by unpaired t test.

**Fig 3:**
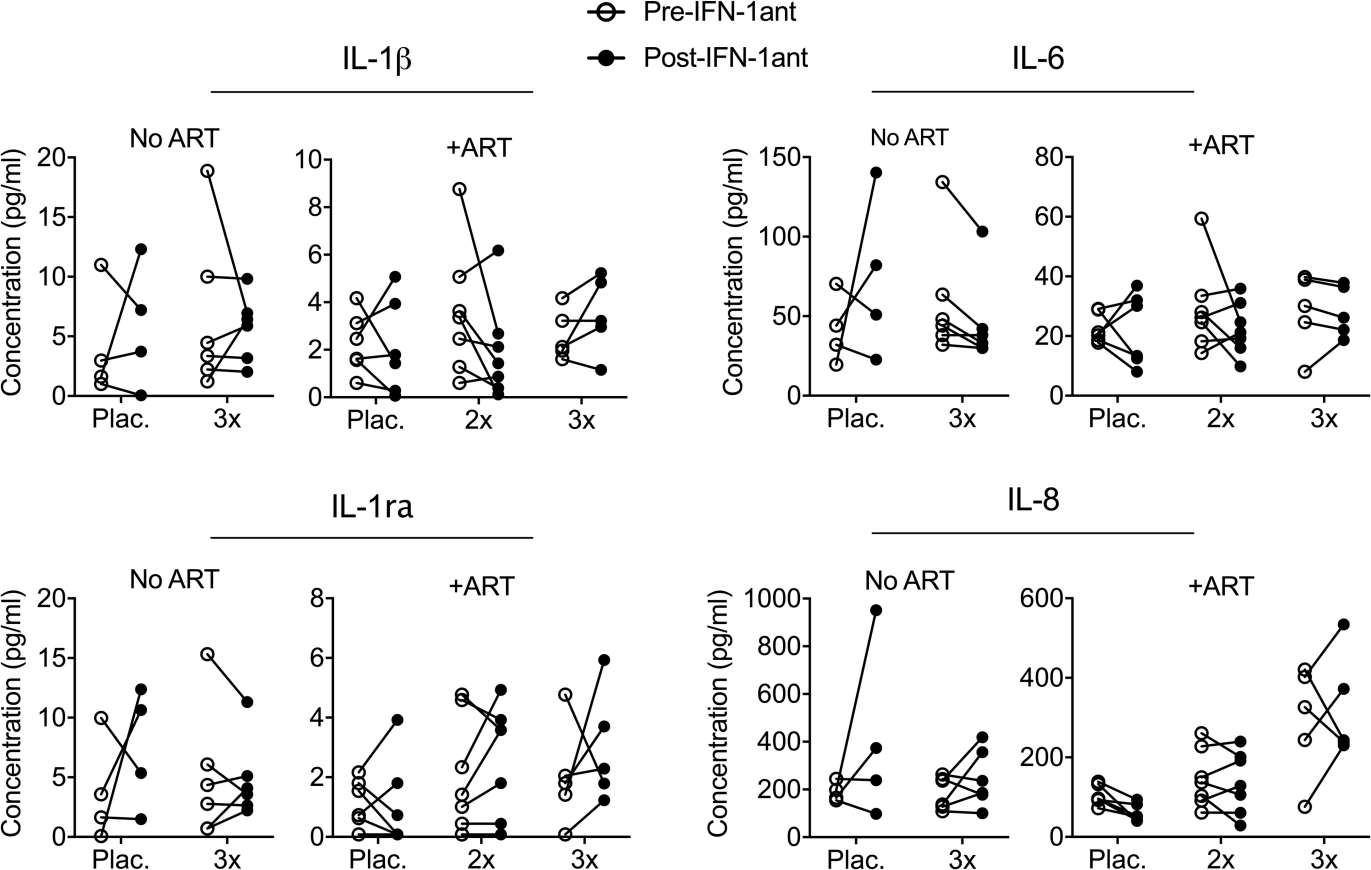
Plasma cytokines. Plasma concentration of IL-1β, IL-1ra, IL-6 and IL-8 before (open circles; week 13) and after (filled circles; week 25) antagonist administration in ART-untreated and ART-treated SIV infected macaques.

We first assessed the longitudinal effects of IFN-I antagonist administration on the expression levels of ISGs at several timepoints: Baseline, post-infection/pre-ART (week 6), pre-IFN-1ant (week 14), during IFN-1ant (weeks 17, 18, 19, 22) and post-IFN-1ant (week 26). For this purpose, we chose a set of 19 ISGs previously demonstrated to be reduced by IFN-1ant treatment during acute SIV infection [5]. As measured by mRNA-seq, the expression levels of several ISGs were increased as expected in all groups following SIV infection (Fig 2A). In the absence of ART, animals treated with IFN-1ant also showed reduction of ISG expression levels compared to placebo animals; but these remained at higher levels in all ART-untreated animals compared to ART-treated animals. In the animals that received ART, there was a significant reduction of ISG expression by week 14, after 6 weeks of ART administration (*P* ≤ 0.0001; S3 Fig). With levels already markedly reduced by ART, administration of the antagonist to these animals from week 16 to week 24 resulted in further reduction of the ISG expression by week 18 and 19 in the 2x weekly treatment group, but were modest in the 3x weekly group (Fig 2A–2C). Quantification of the overall effect of IFN-1ant treatment on ISG expression by comparison of the pre-blockade and post-blockade (wk14 to wk19 post-infection (p.i.) absolute expression levels of the ISG set showed consistent reduction after IFN-1ant treatment in both the 2x weekly ART-treated and ART-untreated animals (Fig 2B). The overall expression of the ISG set showed a 1.9-fold reduction in ART-untreated IFN-1ant animals as compared to placebo (*P* ≤ 0.001; Fig 2C). Among ART-treated animals, 2x weekly administration of the IFN-1ant resulted in a 1.24-fold lower expression of these ISGs (*P* = 0.03) whereas a 3x per week regimen did not result in significant changes (Fig 2C). Overall, the observations of lowered ISG expression after administration of IFN-1ant in the 2x weekly ART group and in the ART-naïve group, demonstrated the pharmacological efficacy of the PASylated IFN-1ant during chronic ART-treated and ART-naïve SIV infection, building on our prior results in which antagonist efficacy was achieved in acute SIV infection. In order to assess the extent of the antagonist-mediated suppression, plasma levels of the cytokines IL-1β, IL-1ra, IL-6 and IL-8 were measured before and after antagonist administration. Despite a significant reduction in ISG expression levels, antagonist treatment in ART-untreated and ART-treated SIV-infected macaques had no effect on circulating levels of these cytokines (Fig 3), suggesting a selective suppression of inflammatory pathways. Collectively, these data demonstrate that IFN-1ant suppressed type I IFN associated inflammatory pathways. Importantly, ART status and viremia impacted on the extent to which antagonism of type I IFN signaling affected expression levels of ISGs. In the absence of ART, ISG expression was reduced but remained higher than when ART alone was given. By itself, ART significantly reduced ISG expression levels and further reduction upon antagonist treatment was more apparent with low viremia.

### Changes in plasma virus load after administration of PASylated IFN-1ant

**Fig 4:**
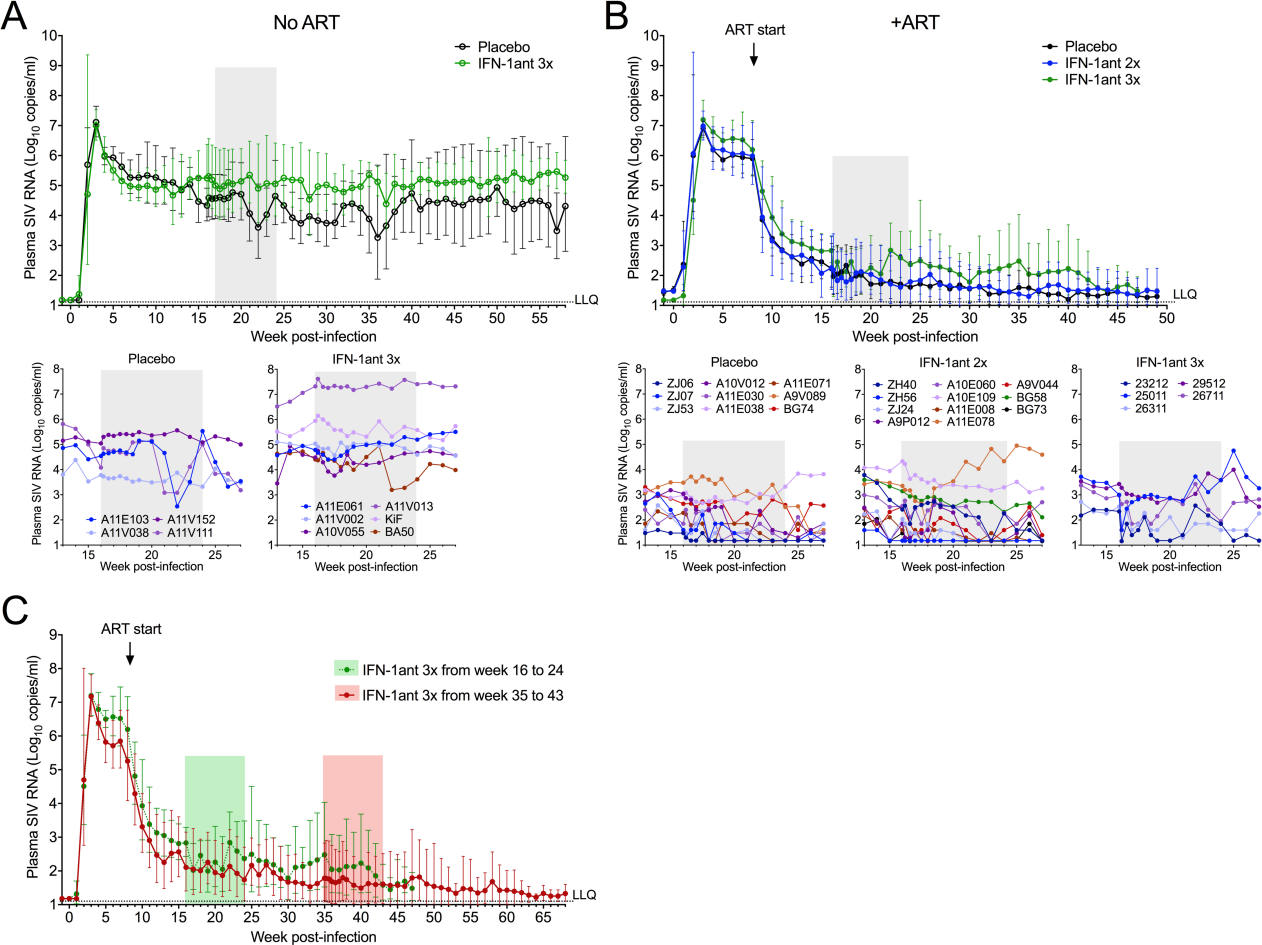
Effect of PASylated IFN-1ant on plasma virus loads. Log_10_ Plasma SIV RNA levels measured from SIV challenge at baseline up to 25 weeks after administration of PASylated IFN-1ant. ART-untreated animals (**a**) received placebo saline (n = 4) or 3 times weekly IFN-1ant injections (n = 6) from week 16 to week 24 p.i. In ART-treated animals (**b**), antiretroviral treatment was initiated at week 8 p.i. and animals received placebo saline (n = 9), IFN-1ant injections 2 times weekly (IFN-1ant2x; n = 11) or 3 times weekly (IFN-1ant3x; n = 5). Shading indicates IFN-1ant treatment period from week 16 to week 24 p.i. Error bars indicate geometric SD and a dotted line indicates the LLQ (lower limit of quantification). Individual plasma virus loads of ART-untreated and ART-treated animals are presented in lower panels. (**c**) Log_10_ Plasma SIV RNA levels measured in macaques challenged with SIV at baseline followed by ART start at week 8 p.i. and finally 3 times weekly administration of PASylated IFN-1ant from week 16 to 24 p.i. (n = 5) or from week 35 to 43 p.i. (n = 5). Shadings indicate IFN-1ant treatment period. Error bars indicate geometric SD and a dotted line indicates the LLQ (lower limit of quantification).

To assess the risk that blocking type I IFN signaling could pose for the control of SIV replication, we measured plasma VL during and after antagonist treatment. In the absence of ART, there was no significant difference between the plasma VL of animals receiving antagonist or placebo from antagonist administration at week 16 p.i. up to week 50 post-infection (Fig 4A). In the ART-treated arm, all groups had measurable plasma VL upon initiation of the antagonist or placebo at week 16 (median plasma VL ranging from 190 to 950 RNA copies/ml) and showed intermittent rebound in plasma VL up to week 49 (Fig 4B). However, from antagonist administration at week 16 until 34 weeks later (i.e. week 50 p.i), pairwise tests of VL or area under the curve showed no significant difference between IFN-1ant-treated animals compared to the placebo group. Regression models adjusting for week 8 (ART start) and week 14 (pre-IFN-1ant) plasma VL also showed no significant effect of the antagonist. Of note, elevated median plasma VL observed from week 20 to 42 in the animals treated 3x per week with antagonist are likely due to the higher VL in these animals prior to initiation of the antagonist treatment. Although statistical significance was not reached, median plasma VL of animals treated 3x per week were consistently higher compared to other ART-treated groups from weeks 10–15 (before antagonist; Fig 4B). To confirm that high plasma VL were unlikely to occur when administering the same IFN-1ant dose regimen to animals having more suppressed viremia, we extended ART treatment and delayed start of the IFN-1ant administration to week 35 instead of 16 in a separate group of animals. In these animals, the virus was further suppressed by week 35 and administration of 3x-weekly antagonist with follow-up for an additional 33 weeks (week 68 p.i) was not associated with plasma VL increases (Fig 4C).

Thus, administration of PASylated IFN-1ant to chronically SIV-infected macaques did not significantly impact plasma VL and appeared to be safe both in ART-treated and untreated infection. Individual plasma VL curves from baseline to week 50 p.i. for all groups are presented in S4 Fig. Given that type I IFN are also important for the control of other viral infections such as CMV, we measured plasma CMV load before and after antagonist administration and observed no increase in both the proportion of CMV^+^ animals or the plasma virus loads of animals that were CMV infected prior to antagonist administration (S1 Table). In addition, administration of the antagonist had no effect on blood chemistry (S2 Table) which is routinely used to gauge major bodily functions. Together, these data reinforce our findings that treatment of SIV-infected macaques with PASylated antagonist, during untreated or ART-suppressed chronic infection, was well tolerated and did not induce overt immunodeficiency.

### Effect of IFN-I blockade on SIV reservoir size

**Fig 5:**
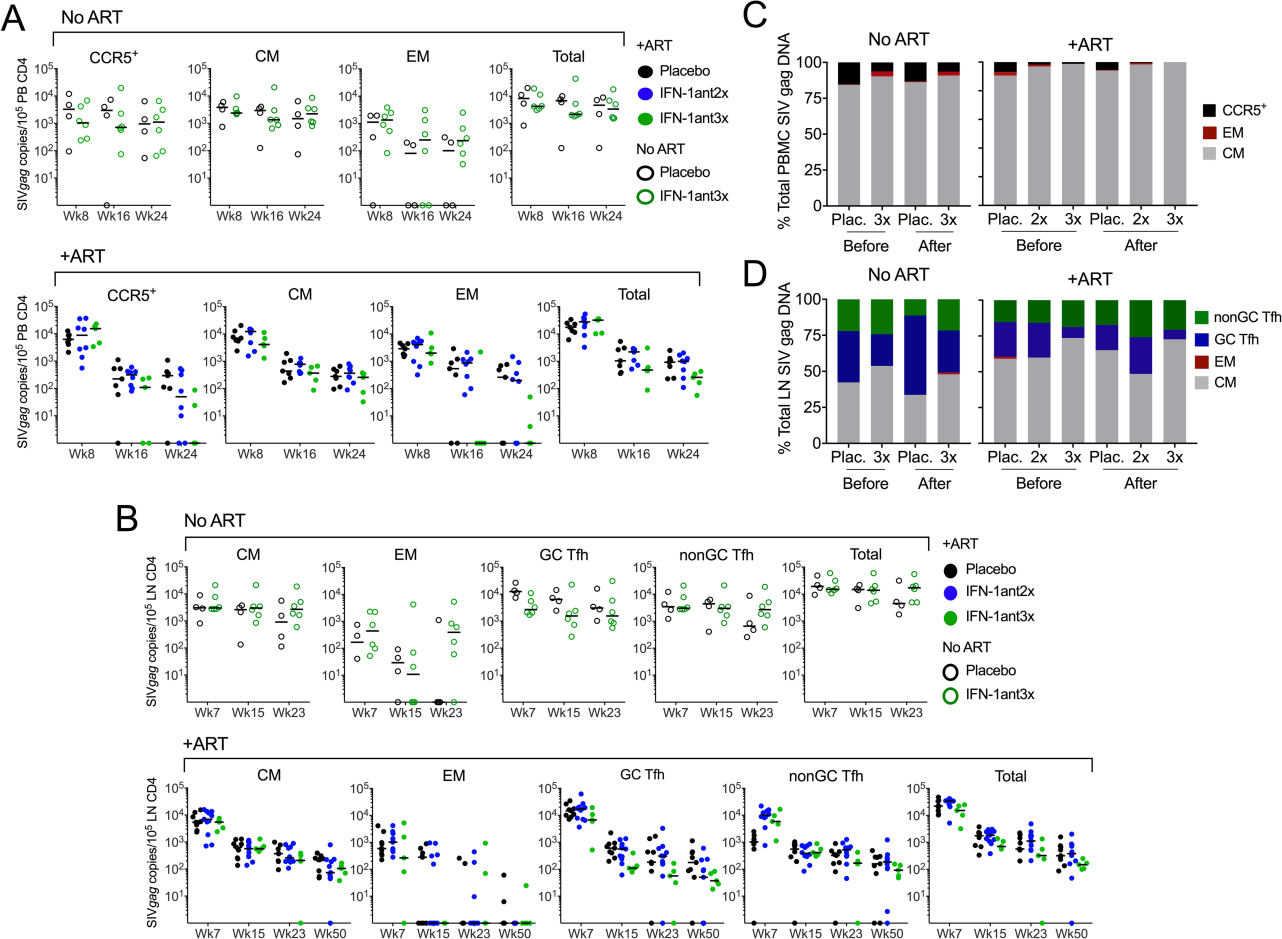
Effect of PASylated IFN-1ant on cell-associated virus. (**a**) PBMC-associated SIV *gag* DNA at week 8 p.i., week 16 p.i. (pre-IFN-1ant) and week 24 p.i. (post-IFN-1ant) in sorted CD4 T cell subsets (CCR5^+^, central memory: CM, effector memory: EM and total) from macaques treated with placebo saline, PASylated IFN-1ant injected 2 times weekly (IFN-1ant2x) or 3 times weekly (IFN-1ant3x). Animals receiving ART starting week 8 p.i. are presented in the lower panel. Horizontal bars represent median values. *P* values were calculated by Mann–Whitney U test. (**b**) LN-associated SIV gag DNA at week 7 p.i., week 15 p.i. (pre-IFN-1ant) and week 23 p.i. (post-IFN-1ant) in sorted CD4 T cell subsets (effector memory: EM, central memory: CM, germinal center T follicular helper: GC Tfh; non-GC Tfh and total) from macaques treated with placebo saline, IFN-1ant injections 2 times weekly (IFN-1ant2x) or 3 times weekly (IFN-1ant3x). Animals receiving ART starting week 8 p.i. are presented in the lower panel. Horizontal bars represent median values. *P* values were calculated by Mann–Whitney U test. (**c**) PBMC and (**d**) LN subset percentages of total SIV *gag* DNA before and after 2 or 3 times weekly IFN-1ant administration in macaques with ART-untreated or ART-treated SIV infection.

We next set out to address whether administration of PASylated type I IFN antagonist impacts the size of the SIV reservoir. Virus DNA was measured in CD4 T cell subsets sorted from PBMC and LN (according to gating strategies shown in S5 Fig). In the PBMC, CD4 T cells were sorted into total CCR5^+^, central memory (CM) and effector memory (EM) cells. While some EM cells express CCR5 [20], all CCR5^+^ cells were gated prior to EM in order to measure virus DNA in the total fraction of cells expressing CCR5 given its role SIV infection. As expected, ART initiation at week 8 significantly reduced the amount of SIV *gag* DNA copies measured in all cell subsets in all animals in each experimental group (Fig 5A). By week 24, treatment with 3x weekly IFN-1ant in ART-treated animals marginally reduced SIV *gag* DNA copies in CCR5^+^ T cells only (*P* = 0.02). However, administration of 2x or 3x weekly IFN-1ant did not show significant effects on CM, EM or total SIV gag copies. In the absence of ART, SIV *gag* DNA copies remained high from week 8 through 24 in PB cell subsets, with no difference between antagonist and placebo groups. In the LN, CD4 T cells were sorted into CM, EM, germinal center follicular helper (GC Tfh) and non-germinal center Tfh (non-GC Tfh) subsets. After ART initiation, the amount of SIV *gag* DNA copies continuously decreased up to week 50 p.i. in all LN subsets in all animals, with no effect of the antagonist treatment given from week 16 to week 24 (Fig 5B). In the absence of ART, the cell-associated SIV *gag* levels remained high across all timepoints and unaffected by IFN-1ant administration in all LN subsets. While cell-associated virus measures are commonly reported using the same numerical denominator, adjusting for the actual frequencies of each subset within a sample gives a more accurate insight into the contribution of each cell subset to the total cell-associated virus DNA level. After adjusting for cell subsets frequencies in our samples, the distribution of SIV *gag* DNA copies across the various PB and LN subsets remained similar between IFN-1ant and placebo treated animals (Fig 5C and 5D). In the PBMC, infected cells belonged almost entirely to the central memory CD4 T cell type (94–100% in ART-treated and 86–91% in ART-untreated). In the LN, infected cells were in majority CD4 CM in ART-treated (up to 71%) and GC Tfh in ART-untreated (up to 55%) animals. We previously observed that type I IFN blockade during untreated, acute SIV infection results in increased reservoir size and accelerated progression to AIDS [5], and raised legitimate concerns regarding safety. In the current data, blocking type I IFN signaling during chronic SIV infection did not increase the size of the SIV virus reservoir irrespective of ART treatment status, even in animals in which residual SIV viremia persisted during ART.

### Effect on T cell activation/exhaustion and T cell phenotypes

**Fig 6:**
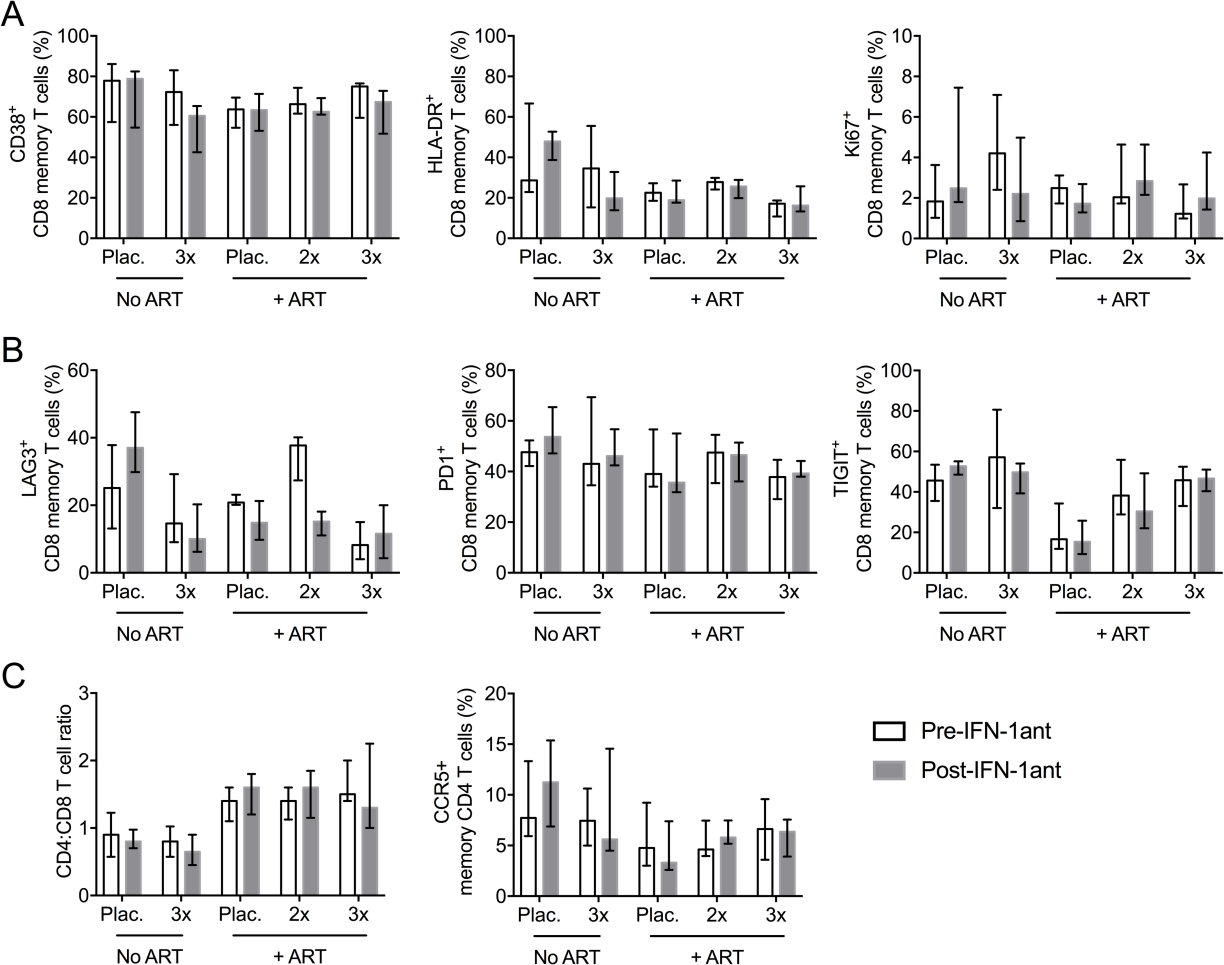
T cell activation/exhaustion and subsets frequencies. (**a**) Peripheral blood frequency of CD38+, HLA-DR+ and Ki67+ CD8 memory T cells, (**b**) Frequency of LAG3+, PD-1+ and TIGIT+ CD8 memory T cells and (**c**) Peripheral blood CD4:CD8 ratio and frequency of peripheral blood CCR5^+^ memory CD4 T cells in ART-untreated or ART-treated macaques that received placebo saline, IFN-1ant injections 2 times weekly (IFN-1ant2x) or 3 times weekly (IFN-1ant3x). Error bars represent IQR. *P* values were calculated between groups by Mann–Whitney U test.

Even though type I IFN antagonist treatment did not significantly affect cell-associated or plasma virus loads, we nevertheless explored its potential effect on modulating T cell function during SIV infection. T cell exhaustion is associated with persistent antigenic stimulation; many reports have previously highlighted that chronic type I IFN signaling during viral infections results in CD8 T cell exhaustion [21–23] and have shown that blocking IFN-I signaling restores T cell function in LCMV-infected mice [13, 14, 24]. Therefore, we assessed T cell activation and exhaustion by measuring frequencies of CD8 memory T cells expressing CD38, HLA-DR, Ki67, LAG-3, PD-1 and TIGIT before and after antagonist administration. None of these markers of T cell activation and exhaustion were affected by antagonist treatment in either ART-untreated or ART-treated SIV infection (Fig 6A and 6B). From our RNA-seq data, comparison of the expression levels of T cell activation and exhaustion genes (S6 Fig) as well as GSEA of T cell activation and exhaustion pathways (S3 Table) showed no significant effect of antagonist treatment. Furthermore, administration of the antagonist did not affect CD4:CD8 ratio and frequencies of CCR5^+^ CD4 T cells in the PBMC (Fig 6C). Therefore, blocking type I IFN signaling during ART-treated or untreated chronic SIV infection showed no significant impact on T cell activation or exhaustion.

### Effect on plasma VL after ART interruption

**Fig 7:**
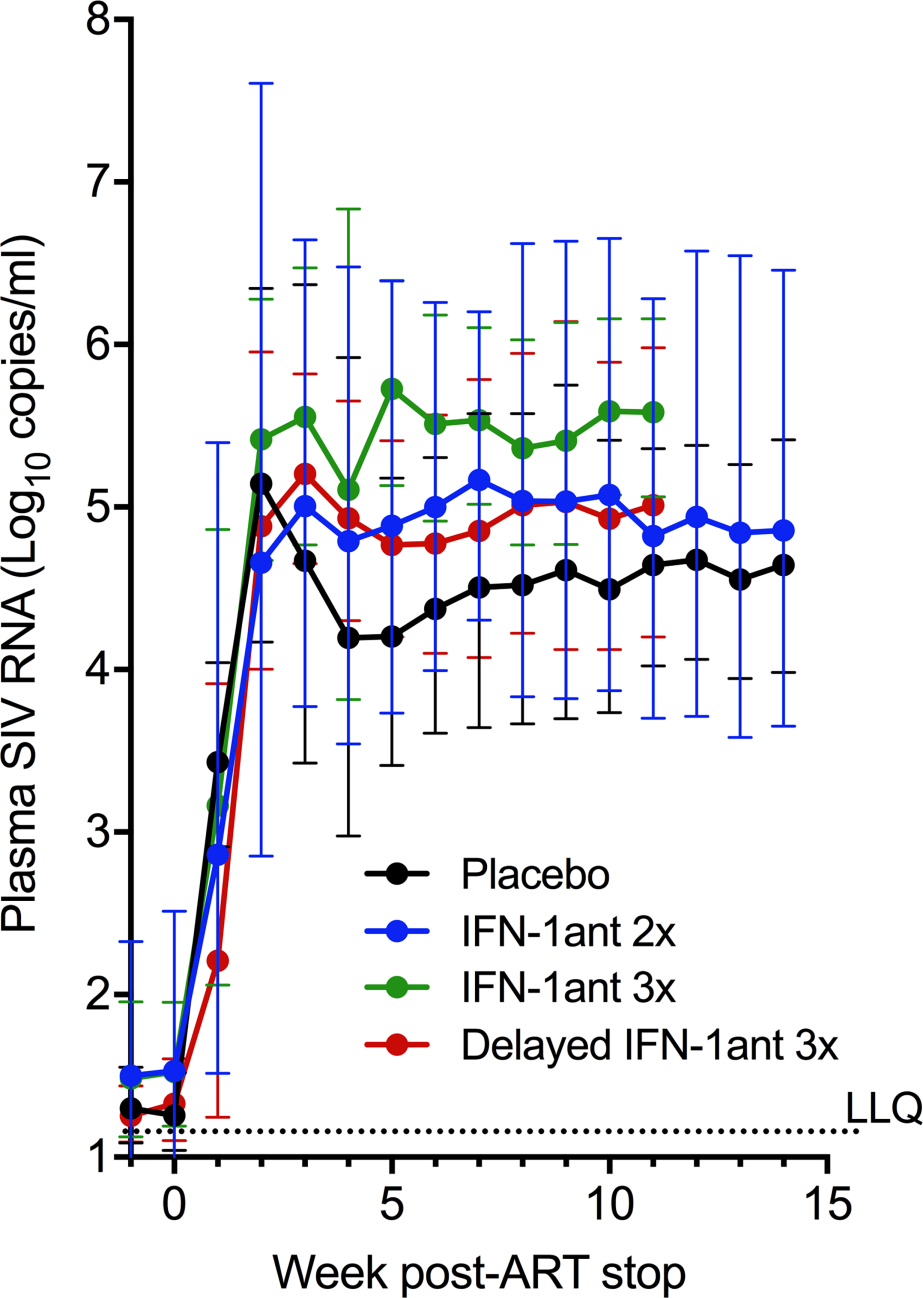
Effect of PASylated IFN-1ant on plasma VL rebound upon ART interruption. Log_10_ Plasma SIV RNA levels measured up to 15 weeks after ART interruption (25 weeks after PASylated IFN-1ant/placebo) in macaques previously treated from weeks 16–24 p.i. with placebo saline (n = 9), IFN-1ant injections 2 times weekly (IFN-1ant2x; n = 11) or 3 times weekly (IFN-1ant3x; n = 5). Delayed IFN-1ant 3x macaques (n = 5) received antagonist treatment from 35–43 p.i. Error bars indicate geometric SD and a dotted line indicates the LLQ (lower limit of quantification).

Finally, we explored whether antagonist administration under ART would influence recrudescence of viremia upon ART interruption. Within a week after ART stop, there was a resurgence of viremia that reached pre-ART levels by week 3 and remained high up to 14 weeks after ART interruption in all groups (Fig 7). Comparison of individual antagonist groups to placebo showed that after adjustment for VL at ART initiation (week 8) there was no significant effect of the antagonist on the mean log_10_ plasma VL set point defined as 6 consecutive weeks starting 4 weeks after ART cessation. Thus, despite significantly reducing expression of ISGs, blocking type I IFN signaling in ART-treated chronic SIV infection did not result in increased plasma VL compared to placebo even after ART interruption.

## Discussion

Our findings are primarily relevant to the implementation of IFN-I blockade strategies in clinical HIV studies. Type I IFNs are important mediators of antiviral immunity but their permanent engagement in chronic HIV infection also contributes to a persistent inflammatory state that promotes pathology. As is being advocated for inflammatory diseases such as systemic lupus erythematous or systemic sclerosis [12], therapeutic blockade of IFN-I signaling could reduce inflammation and improve control of HIV infection. However, adverse outcomes observed in acute SIV infection [5] raised major safety concerns for the potential use of similar approaches during the chronic stage. Here, we assessed the effect of blocking IFN-I signaling during ART-treated and ART-untreated chronic SIV infection. Our principal findings were: (1) administration of an IFN-I receptor antagonist with prolonged half-life to ART-treated and ART-untreated SIV-infected rhesus macaques showed a therapeutic benefit in terms of lowering inflammation in part as observed by amelioration of ISG expression despite unaltered levels of measured pro-inflammatory cytokines; and (2) in contrast to observations made during acute SIV infection [5], blockade of IFN-I signaling during chronic SIV infection did not lead to loss of control of viral replication. In this regard, our study shows that in chronic SIV infection, even in situations with residual viral replication, blocking type I IFN signaling did not lead to loss of control of the infection; and supports the rationale that the use of an IFN-I antagonist during chronic HIV infection is safe.

The difference in outcome of IFN-I signaling blockade between acute [5] and chronic SIV infection is most likely due to the timing of IFN-I signaling for control of the infection. Significant increase in plasma VL and SIV reservoir size upon blockade of IFN-I signaling in the acute phase implies a critical role for IFN-I at the onset of infection. In contrast, our data show that once persistent infection has been established, IFN-I signaling plays a less prominent role in the control of virus replication. Recently, administration of exogenous IFN-I along with ART to chronically SIV infected animals was shown to increase ISGs expression with no effect on virus control [25]. Similar differences in the role of IFN-I between acute and chronic viral infection have been reported in LCMV studies. For instance, addition of exogenous IFN-I increases control of the infection in the acute stage but does not decrease virus titers in the chronic stage [22, 26]. While such findings support the notion that the importance of IFN-I signaling for viral control changes over the course of the infection, the underlying reasons remain unclear.

It remains uncertain what clinical benefits would emerge as a result of blocking IFN-I signaling during chronic HIV infection. In ART-treated HIV infection, IFN-I signatures remain elevated despite effective HIV suppression by cART [27]. Targeting IFN-I could further suppress residual inflammation and rescue T cells from exhaustion. Despite reduced expression of some of the most prominent antiviral ISGs downstream of IFNAR such as *Mx1* and *OAS2* [28], blocking IFN-I signaling in our study had no measurable impact on T cell activation or exhaustion. Of note, the higher antagonist dose (3x weekly) in our study was less efficacious in reducing ISG expression, which may be due to partial agonist activity of the antagonist to induce minimal ISG expression, which has been reported at high concentrations *in vitro* [29]. In addition, because the 3x weekly ART group had a generally higher VL compared to animals that received twice weekly IFN-1ant (Fig 4), there is likely a threshold where any measurable effect of the antagonist added to the effect of ART is influenced by the viremia at the time antagonist treatment is initiated. Our findings possibly reinforces the importance of timing on the outcome of IFN-I signaling blockade as administration of IFN-I antagonist [5] and more recently anti-IFN antibody treatment [30] in SIV infected macaques was shown to reduce T cell activation. The observations in our study also differ from observations made in prior studies on IFN-I blockade in chronically HIV-infected humanized mice which showed reduced expression of ISGs along with enhanced viral suppression and reduced T cell activation [15, 16]. There are notable differences in experimental design that could account for this discrepancy. With a maximum of 4 weeks antibody treatment in the mice as opposed to 8 weeks of a long half-life IFN-I antagonist in our study, it is possible that both timing and duration could influence the outcome of blocking IFN-I signaling. Blockade for a limited time may rescue immune responses but viral clearance mechanisms may require a contribution from the IFN-I signaling pathway. Another possibility may be related to the animal models used. While the studies in humanized mice indicate that a reduction of cellular makers of inflammation was associated with improved control of virus replication, our data in non-human primates suggest that a lengthy blockade of IFN-I signaling reduces ISG expression but has no effect on other inflammatory pathways or T cell activation and thus may not affect control of virus replication. The important point, however, is that blockade of IFN-I signaling in chronically SIV-infected non-human primates did not lead to an *increase* in SIV replication as is the case in acute SIV infection. Dissection of experimental variables such as timing, duration, dose and possibly the evaluation of combination interventions in future studies will delineate optimal conditions for the therapeutic blockade of IFN-I signaling. A recent study in LCMV demonstrated that virus control and T cell exhaustion are mediated by different type I IFNs despite their use of the same receptor [24] and HIV studies on administration of various IFN-I subtypes to humanized mice revealed differences in their ability to suppress the virus [31, 32]. Thus, an intriguing hypothesis is that by manipulating specific type I IFNs and their subtypes (i.e. IFNα vs. IFNβ) in HIV/SIV infection, it may be possible to decouple the various biological activities of the IFN-system and selectively target deleterious activities while maintaining beneficial ones.

Administration of antiretrovirals in our study successfully suppressed SIV but most animals had virus loads above the limit of detection even after 40 weeks of ART. Consequently, the antagonist was administered to animals with detectable plasma VL. The presence of this residual viremia allowed evaluation of the safety aspect of IFN blockade in chronic SIV infection, which was a concern in light of previous findings that blockade during acute infection accelerated mortality. While our study concluded that IFN-I blockade in chronic SIV infection did not impair control of the infection, it remains to be seen whether the pre-intervention plasma virus load influences the outcome of IFN-I blockade. The use of, for example, a macrophage-tropic virus to assess how IFN-blockade influences infection of myeloid cells will help gain a full understanding of the clinical implications of blocking IFN-I in chronically HIV-infected persons.

Despite reductions in risk of death with ART, high rates of serious non-AIDS events associated with inflammation [3] continue to reduce quality and expectancy of life in HIV-infected people [33, 34]. Thus, therapeutic blockade of IFN-I signaling which plays a key role in the persistence of inflammation, even during suppressive ART, has the potential to safely improve clinical outcome in HIV-infected persons.

## Materials and methods

### Animals and experimental design

40 Mamu A01^-^ B08^-^ B17^-^ adult Indian origin *Macaca mulatta* were challenged by two intrarectal inoculations within five days of 1ml SIV_MAC251_ (1ml of 1:25 dilution, stock 8.91 x 10^8^ SIV RNA copies ml^-1^). At week 8 post-infection, 30 animals were started on antiretrovirals while 10 animals were left untreated. The ART regimen was subcutaneous injections of 20mg/kg/day Tenofovir and 30mg/kg/day Emtricitabine (Gilead), as well as orally administered drugs including 100mg BID Raltegravir (Merck), 800mg BID Darunavir (Janssen Pharmaceuticals) and 100mg BID Ritonavir (Abbvie), all given mixed with food. To investigate the effect of blocking IFN-I signaling, infected animals received i.m. injection of 3.5mg per injection given 2 or 3 times per week with a type I interferon receptor antagonist (IFN-1ant) used previously [5] whose plasma half-life was significantly increased by PASylation [19] as assessed in healthy macaques. PAS-IFN-1ant was produced by fermentation in *E*. *coli* according to a published procedure [19] where a human IFN-α2b carrying the amino acid substitutions R120E, E159K, S160R and R162K (in the mature protein) was equipped with a structurally disordered N-terminal PAS#1 sequence of 600 residues and secreted into the bacterial periplasm to facilitate formation of the structural disulfide bonds. Purification was achieved by substractive anion exchange chromatography and ammonium sulfate precipitation followed by a cation exchange and a finishing anion exchange chromatography, resulting in a homogenous protein preparation with ≤5.5 IU endotoxin per mg protein. Based on plasma concentrations measured after *in vivo* administration of the antagonist to healthy macaques, the chosen regimen of 2 and 3 times per week should result in minimum plasma concentrations of 5 nM and 10–20 nM respectively (S1 Fig). Detailed experimental set-up and IFN-1ant treatment is presented in Fig 1.

### Ethics statement

Animal use and all study procedures (protocol VRC-13-453, renewed once as VRC-16-678) were approved by the Vaccine Research Center (VRC) Animal Care and Use Committee (ACUC), meeting National Institutes of Health standards; and in accordance with the American Association for Accreditation of Laboratory Animal Care guidelines, all federal, state, and local regulations, and in compliance with *The Guide for the Care and use of Laboratory Animals*. All animals, Indian origin rhesus macaques (*Macaca mulatta*) were socially housed at the National Institutes of Health with oversight from facility behavioral management staff. Primary enclosures consisted of stainless steel primate caging provided by a commercial vendor. Animal body weights and cage dimensions were regularly monitored. Overall dimensions of primary enclosures (floor area and height) met the specifications of The Guide for the Care and Use of Laboratory Animals, and the Animal Welfare Regulations (AWR's). All primary enclosures were sanitized every 14 days at a minimum, in compliance with AWRs. Secondary enclosures (room level) met specifications. All animals were housed under controlled conditions of humidity, temperature and light (12-hour light/12-hour dark cycles). Animals were fed commercial monkey chow, twice daily, with supplemental fruit or other produce at least three times per week. Filtered water was available ad libitum. Animals were observed at least twice daily by trained personnel, including behavioral assessments. Environmental enrichment included provision of species appropriate manipulatives, and foraging opportunities, as well music and video watching opportunities multiple times per week. No adverse events have been associated with study interventions. For procedures requiring chemical immobilization and sedation, different anesthetics were used at the discretion of the attending veterinarian according to the IACUC approved protocol. Prior to immunization, drug treatments or blood draws, anesthetics included Ketamine Hydrochloride 5.0–25.0 mg/kg IM with xylazine 0.5–1.0 mg/kg. For technical procedures, Buprenorphine Hydrochloride 0.015 mg/kg was administered. For euthanasia according to endpoints specified in the IACUC approved protocol, animals were initially sedated with ketamine (10–25 mg/kg, IM) followed by lethal overdose of sodium pentobarbital to effect.

### Blood and lymph node samples processing

Plasma was separated from EDTA blood by centrifugation and PBMCs were isolated by density centrifugation using Ficoll-Paque Plus (GE Healthcare) and Leucosep Centrifuge Tubes (Grenier Bio-One). Lymph nodes (LN) were collected into RPMI 1640 (Gibco) supplemented with 10% fetal bovine serum (Gibco) and 1% Penicillin-Streptomycin-Glutamine (Gibco) and cell suspensions were passed through a 70μm filter to remove debris.

### Flow cytometry and cell sorting

For CD4 T cell subsets sort, PBMC were stained with fluorochrome-labelled mAbs anti-CD28-CY5PE, anti-CCR5-PE, anti-CD3-CY7APC, anti-CD4-BV605, anti-CD8-Pacific blue (BD Biosciences) and anti-CD95-BV785 (in house conjugated, BD Biosciences). LN cells were stained with anti-CD28-CY5PE, anti-CD3-CY7APC, anti-CD4-BV605 (BD Biosciences), anti-CD8-BV570, anti-CXCR5-PE (eBioscience) and anti-CD95-BV785 (in house conjugated, BD Biosciences). PBMC and LN CD4 subsets of interest were sorted and lyzed in proteinase K (100ug mL^-1^, Sigma Aldrich) for SIV *gag* qPCR. For assessment of T cell activation and exhaustion, cryopreserved PBMC were thawed and stained with fluorochrome-labelled mAbs anti-CD38-FITC (Stem Cell), anti-Ki67-CY7PE, anti-CD28-CY5PE, anti-CD3-CY7APC (BD Biosciences), anti-HLA-DR-TRPE (Life Technologies), anti-PD-1-BV711, anti-CD95-BV785 (Biolegend), anti-TIGIT-APC (ThermoFisher) and anti-LAG3-PE (R&D). All samples were stained with Aqua LIVE/DEAD Fixable Dead Cell Stain.

### Plasma viral load and cell associated viral load measurement

Plasma SIV*gag* RNA was assayed as described previously [35]. For cell associated virus, SIV*gag* and rhesus albumin DNA were simultaneously quantified in cell lysates by qPCR using plasmid standards for absolute quantification of *gag* and albumin copy numbers with the following primers and probes used at final concentrations of 625nM and 250nM, respectively:

SIV-Gag-F    GTCTGCGTCATpTGGTGCATTC

SIV-Gag-R    CACTAGkTGTCTCTGCACTATpTGTTTTG

SIV-Gag-P    CTTCpTCAGTkTGTTTCACTTTCTCTTCTGCG

Rh-Alb-F      TGCATGAGAAAACGCCAGTAA

Rh-Alb-R      ATGGTCGCCTGTTCACCAA

Rh-Alb-P      AGAAAGTCACCAAATGCTGCACGGAATC

### Plasma cytokines

Plasma concentrations of IL-1β, IL-1ra, IL-6 and IL-8 were measured by bioplex assay using a premixed non-human primate kit from Millipore according to the manufacturer recommendations.

### CMV testing

DNA was isolated from plasma using the QIAamp DNA Blood Mini Kit (QIAgen 51106) according to manufacturer’s instructions. Isolated DNA was then analyzed using an Applied Biosystems QuantStudio Real-Time PCR system (12K Flex) with Cytomegalovirus (CMV) specific primers (Forward: ATC CGC GTT CCA ATG CA, Reverse: CGG AGG AGC ACC ATA GAA GGT) and a TaqMan Probe (6FAM CCT TCC CGG CTA TGG MGBNFQ). Each sample was run in triplicate for 40 cycles along with positive controls. Copy number was calculated by comparison to a standard curve and the viral load was reported as CMV copies/mL of plasma.

### Transcriptome measurement and analysis

RNA was extracted from cryopreserved PBMCs using RNAzolRT (Molecular Research Center) according to the manufacturers’ instructions. Purified RNA was used for transcriptome analysis. Briefly, polyadenylated transcripts were purified on oligo-dT magnetic beads, fragmented, reverse transcribed using random hexamers and incorporated into barcoded cDNA libraries based on the Illumina TruSeq platform. Libraries validated by microelectrophoresis were sequenced on an Illumina HiSeq 4000 in 100-base single-read reactions. RNA-seq analysis was conducted at the Yerkes Nonhuman Primate Genomics Core Laboratory. Estimates of gene-wise and isoform-wise expression levels for individual genes were performed using DESeq2 [36]. The RNA-seq data were submitted to the Gene Expression Omnibus repository at the National Center for Biotechnology Information database (GSE112148).

To identify pathways differentially modulated by IFN-1ant, Gene Set Enrichment Analysis was performed as follows. For each contrast, transcripts were ranked by differential expression using the Signal2Noise metric. GSEA was performed using the desktop module available from the Broad Institute (www.broadinstitute.org/gsea/). GSEA was performed on the ranked transcript lists using 1,000 phenotype permutations, and random seeding. Gene sets used included the MSigDB (http://software.broadinstitute.org/gsea/msigdb/collections.jsp) H (hallmark), C5 (GO), C2 (curated), C7 (immunologic) gene sets ([37]), and additional custom gene sets.

## Supporting information

S1 Fig Pharmacokinetic properties of PASylated IFN-1ant. (**a**) Plasma concentrations of IFN-I as measured by ELISA in healthy macaques following a single i.m. injection with either 1 mg (49.8 nmol) unmodified IFN-1ant (N = 4) or 3.5 mg (51.5 nmol) of PASylated IFN-1ant (PAS-IFN1-ant; N = 4). Based on PK parameters measured for unmodified IFN-1ant and its PASylated version, plasma concentration profiles were simulated for both proteins according to a 2 or 3 times per week dosing schemes; showing a minimum plasma concentration of 5nM in a 2x per week regimen (**b**), 10-20nM in a 3x per week regimen (**c**) and both superior to the unmodified antagonist.
(TIF)

S2 Fig Plasma virus loads before ART and prior to antagonist treatment. Log_10_ Plasma SIV RNA levels at weeks 4–5, 8 and 14 p.i. in animals treated from week 16–24 p.i. with placebo saline or PASylated IFN-1ant 2 times per week (IFN-1ant 2x) or 3 times per week (IFN-1ant 3x). ART-treated animals started antiretrovirals on week 8 p.i. Horizontal lines indicate medians.
(TIF)

S3 Fig Effect of ART on ISG expression levels. Fold change of the median read counts of the 19 ISGs in ART-treated and ART-untreated macaques from week 6 to week 14 p.i. Error bars indicate lower and upper quartiles. *P* values were calculated by unpaired t test.
(TIF)

S4 Fig Individual plasma virus loads from baseline until week 50 p.i. Log_10_ Plasma SIV RNA levels measured from SIV challenge at baseline up to 25 weeks after administration of PASylated IFN-1ant. ART-untreated animals received placebo saline (n = 4) or 3 times weekly IFN-1ant injections (n = 6). In ART-treated animals, antiretroviral treatment was initiated at week 8 p.i. and animals received placebo saline (n = 9), IFN-1ant injections 2 times weekly (IFN-1ant2x; n = 11) or 3 times weekly (IFN-1ant3x; n = 5). Shading indicates IFN-1ant treatment period from week 16 to week 24 p.i.
(TIF)

S5 Fig Gating strategy sorted CD4 T cell subsets. (**a**) PBMC CD4 T cells were sorted into CCR5^+^ defined as Aqua^-^CD3^+^CD8^-^CD4^+^CCR5^+^, central memory (CM) defined as Aqua^-^CD3^+^CD8^-^CD4^+^CCR5^-^CD28^+^CD95^+^ and CCR5^-^ effector memory (EM) defined as Aqua^-^CD3^+^CD8^-^CD4^+^CCR5^-^CD28^+^CD95^-^. (**b**) LN CD4 T cells were sorted into effector memory (EM) defined as Aqua^-^CD3^+^CD8^-^CD4^+^CD28^+^CD95^-^, central memory (CM) defined as Aqua^-^CD3^+^CD8^-^CD4^+^CD28^+^CD95^+^CXCR5^lo^PD-1^lo^, germinal center T follicular helper (GC Tfh) defined as Aqua^-^CD3^+^CD8^-^CD4^+^CD28^+^CD95^+^CXCR5^hi^PD-1^hi^ and non-GC Tfh defined as Aqua^-^CD3^+^CD8^-^CD4^+^CD28^+^CD95^+^CXCR5^int^PD-1^int^.
(TIF)

S6 Fig T cell activation and exhaustion genes heatmaps. Heatmaps indicate expression of select genes associated with T cell activation, or exhaustion/inhibition at time points prior to (Week 14 post-infection) and following (Week 19 post-infection) IFN-1ant treatment. Heatmap colors represent gene expression levels that were log_2_ transformed (Variance stabilizing transformation, DESeq2) and normalized to the Week 14 median expression level of each transcript. Statistical significance of differences between Week 14 and Week 19 expression levels for each transcript was determined using negative binomial generalized linear models (DESeq2).
(TIF)

S1 Table CMV plasma virus load before and after administration of PASylated antagonist. (DOCX)

S2 Table Blood chemistry before and after administration of PASylated antagonist. (DOCX)

S3 Table T cell Exhaustion and Activation GSEA FDR q-values. (DOCX)
