# Supplementary figures and images for: Type I IFN signaling blockade by a PASylated antagonist during chronic SIV infection suppresses specific inflammatory pathways but does not alter T cell activation or virus replication
Source: PLoS Pathog. 2018 Aug 24;14(8):e1007246. doi: 10.1371/journal.ppat.1007246 (PMC6126880; doi:10.1371/journal.ppat.1007246)

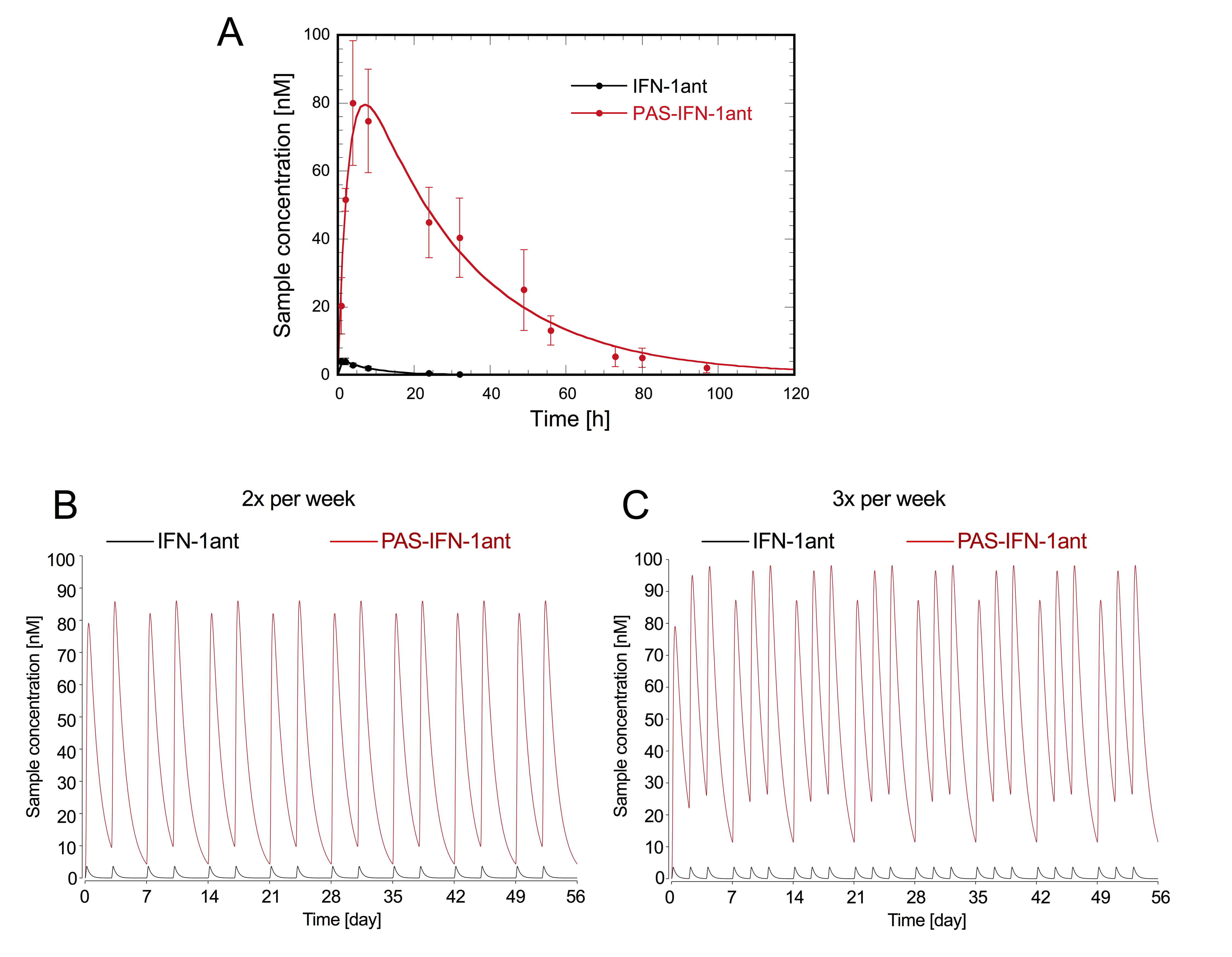

Supplement: S1 Fig — (a) Plasma concentrations of IFN-I as measured by ELISA in healthy macaques following a single i.m. injection with either 1 mg (49.8 nmol) unmodified IFN-1ant (N = 4) or 3.5 mg (51.5 nmol) of PASylated IFN-1ant (PAS-IFN1-ant; N = 4). Based on PK parameters measured for unmodified IFN-1ant and its PASylated version, plasma concentration profiles were simulated for both proteins according to a 2 or 3 times per week dosing schemes; showing a minimum plasma concentration of 5nM in a 2x per week regimen (b), 10-20nM in a 3x per week regimen (c) and both superior to the unmodified antagonist. (TIF) [file ppat.1007246.s001.tif]

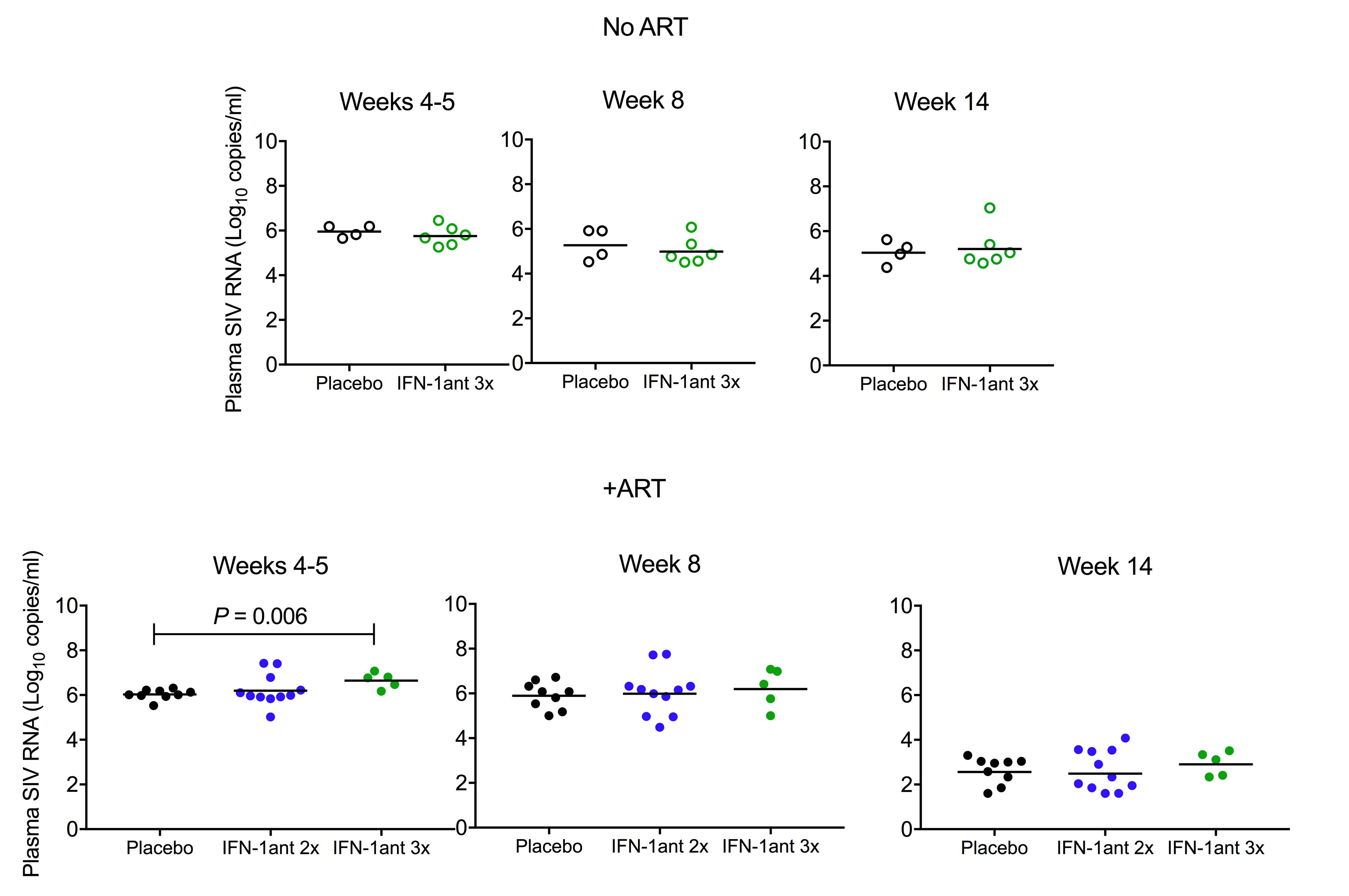

Supplement: S2 Fig — Log10 Plasma SIV RNA levels at weeks 4–5, 8 and 14 p.i. in animals treated from week 16–24 p.i. with placebo saline or PASylated IFN-1ant 2 times per week (IFN-1ant 2x) or 3 times per week (IFN-1ant 3x). ART-treated animals started antiretrovirals on week 8 p.i. Horizontal lines indicate medians. (TIF) [file ppat.1007246.s002.tif]

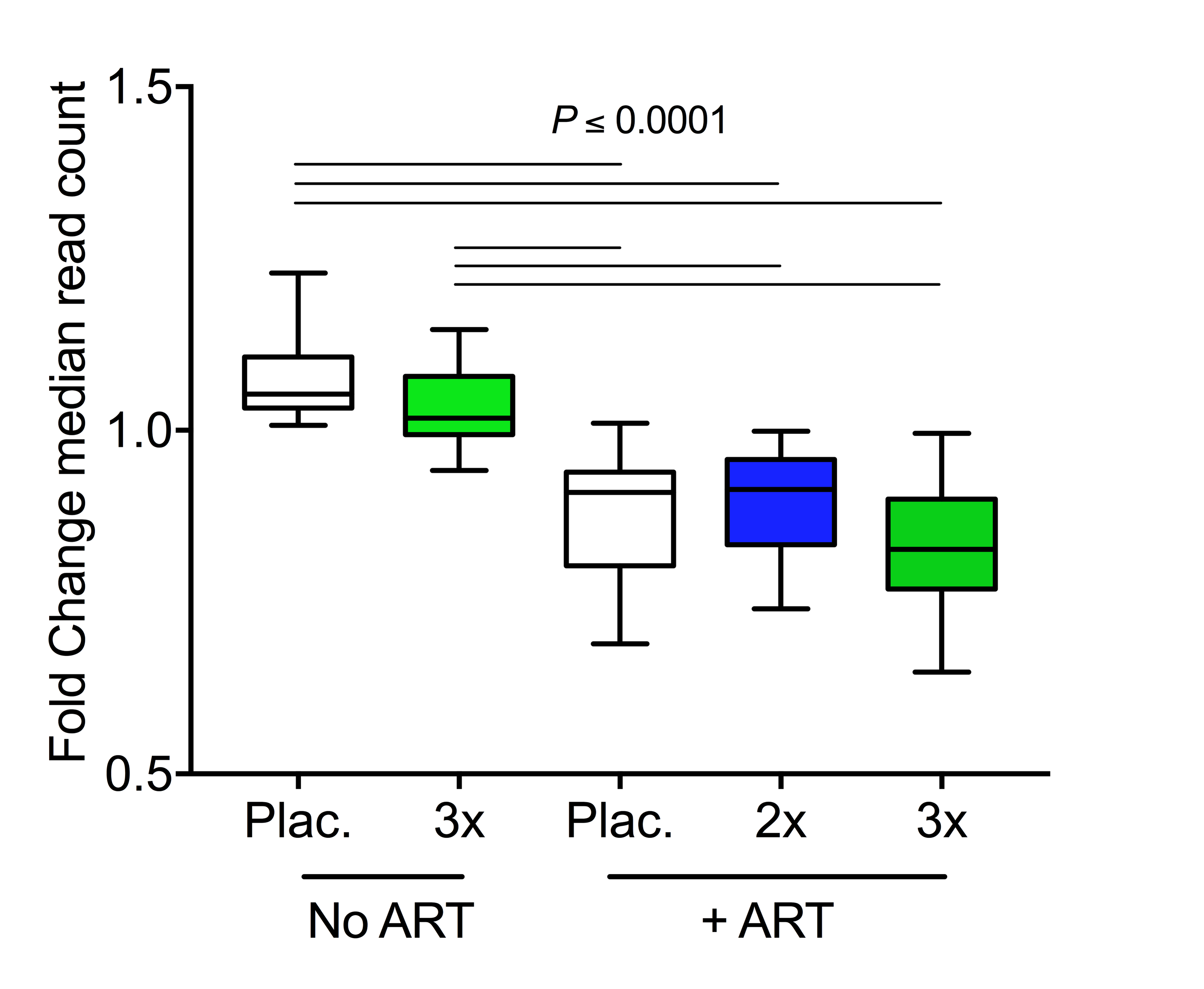

Supplement: S3 Fig — Fold change of the median read counts of the 19 ISGs in ART-treated and ART-untreated macaques from week 6 to week 14 p.i. Error bars indicate lower and upper quartiles. P values were calculated by unpaired t test. (TIF) [file ppat.1007246.s003.tif]

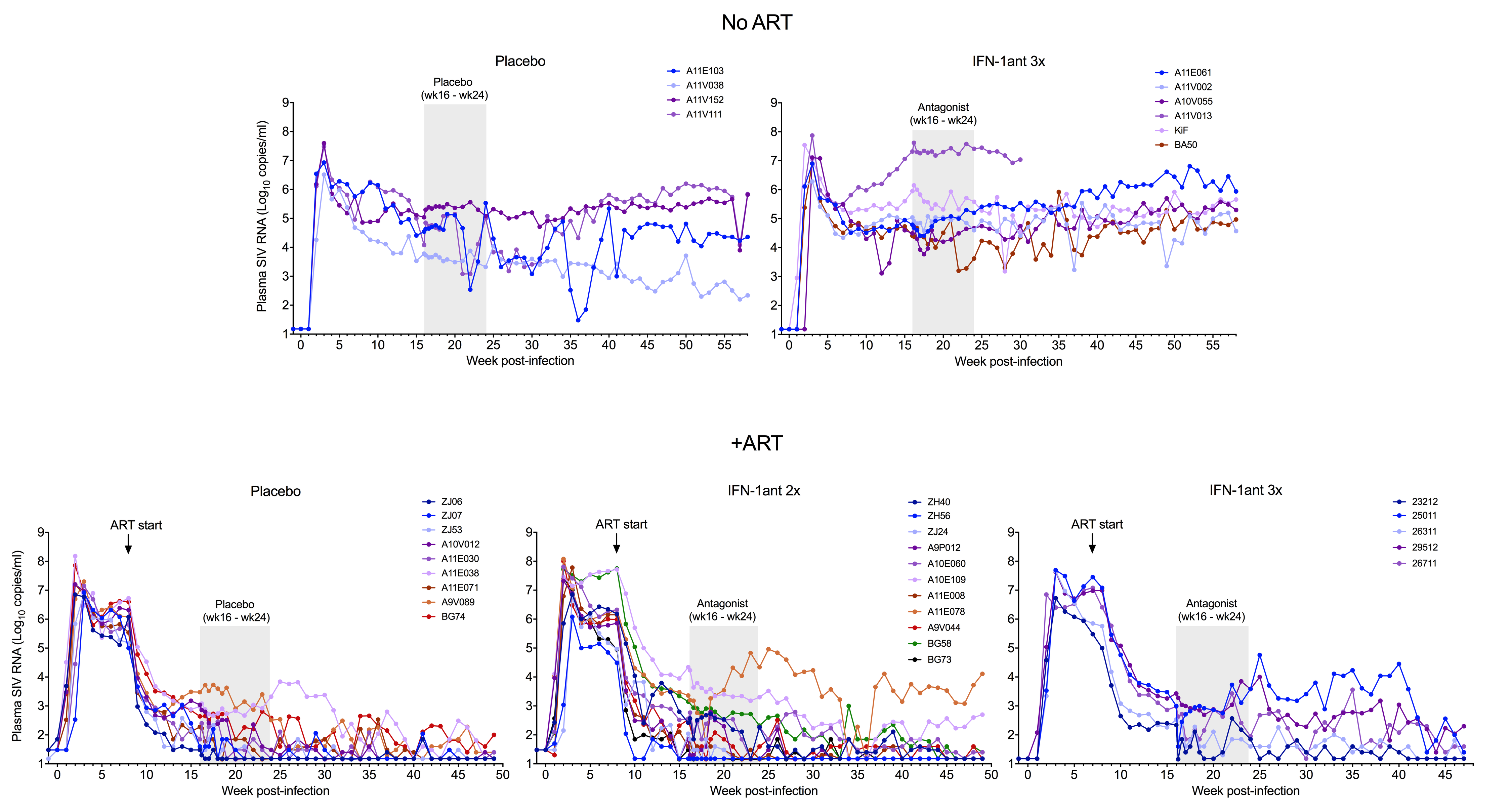

Supplement: S4 Fig — Log10 Plasma SIV RNA levels measured from SIV challenge at baseline up to 25 weeks after administration of PASylated IFN-1ant. ART-untreated animals received placebo saline (n = 4) or 3 times weekly IFN-1ant injections (n = 6). In ART-treated animals, antiretroviral treatment was initiated at week 8 p.i. and animals received placebo saline (n = 9), IFN-1ant injections 2 times weekly (IFN-1ant2x; n = 11) or 3 times weekly (IFN-1ant3x; n = 5). Shading indicates IFN-1ant treatment period from week 16 to week 24 p.i. (TIF) [file ppat.1007246.s004.tif]

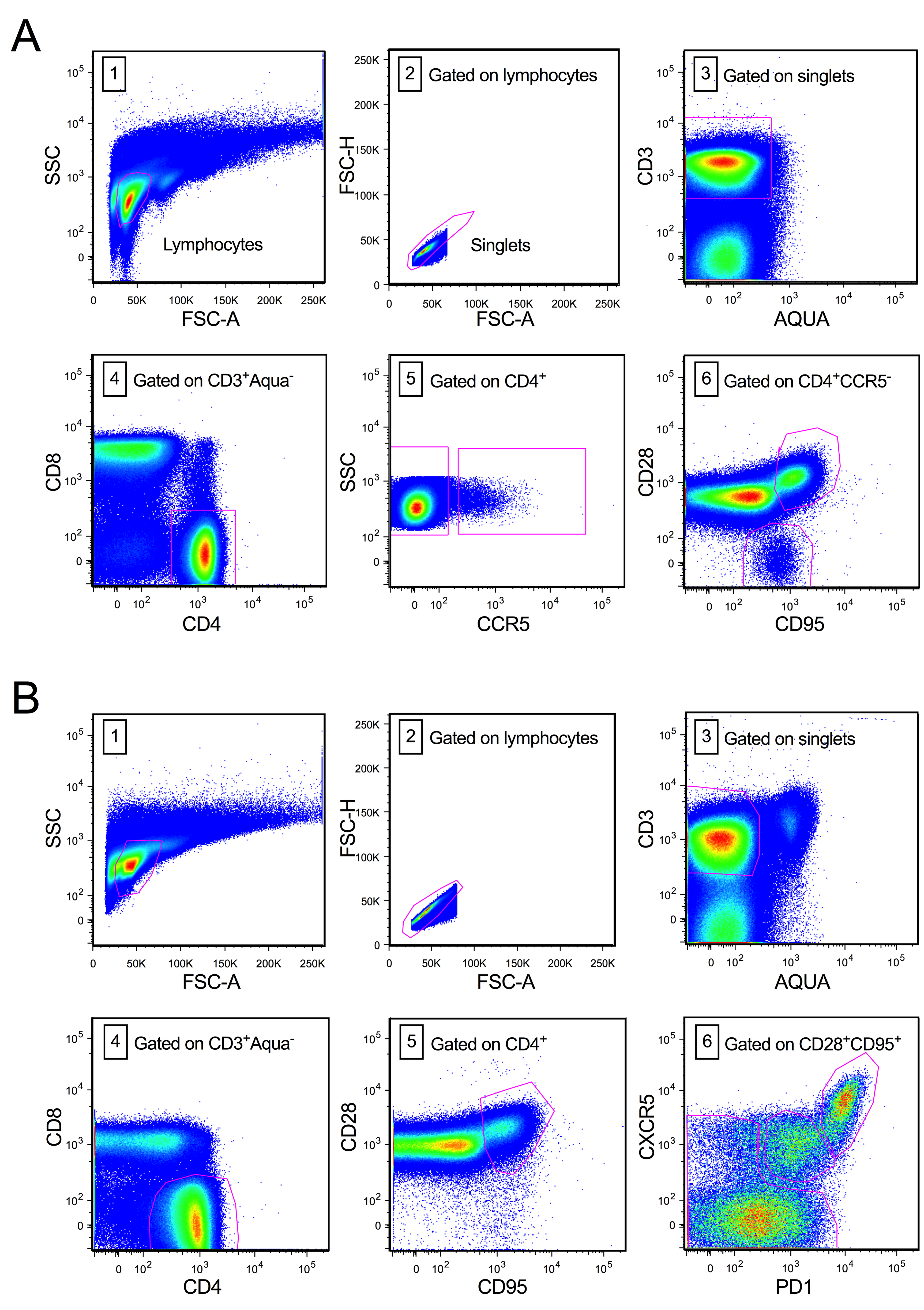

Supplement: S5 Fig — (a) PBMC CD4 T cells were sorted into CCR5+ defined as Aqua-CD3+CD8-CD4+CCR5+, central memory (CM) defined as Aqua-CD3+CD8-CD4+CCR5-CD28+CD95+ and CCR5- effector memory (EM) defined as Aqua-CD3+CD8-CD4+CCR5-CD28+CD95-. (b) LN CD4 T cells were sorted into effector memory (EM) defined as Aqua-CD3+CD8-CD4+CD28+CD95-, central memory (CM) defined as Aqua-CD3+CD8-CD4+CD28+CD95+CXCR5loPD-1lo, germinal center T follicular helper (GC Tfh) defined as Aqua-CD3+CD8-CD4+CD28+CD95+CXCR5hiPD-1hi and non-GC Tfh defined as Aqua-CD3+CD8-CD4+CD28+CD95+CXCR5intPD-1int. (TIF) [file ppat.1007246.s005.tif]

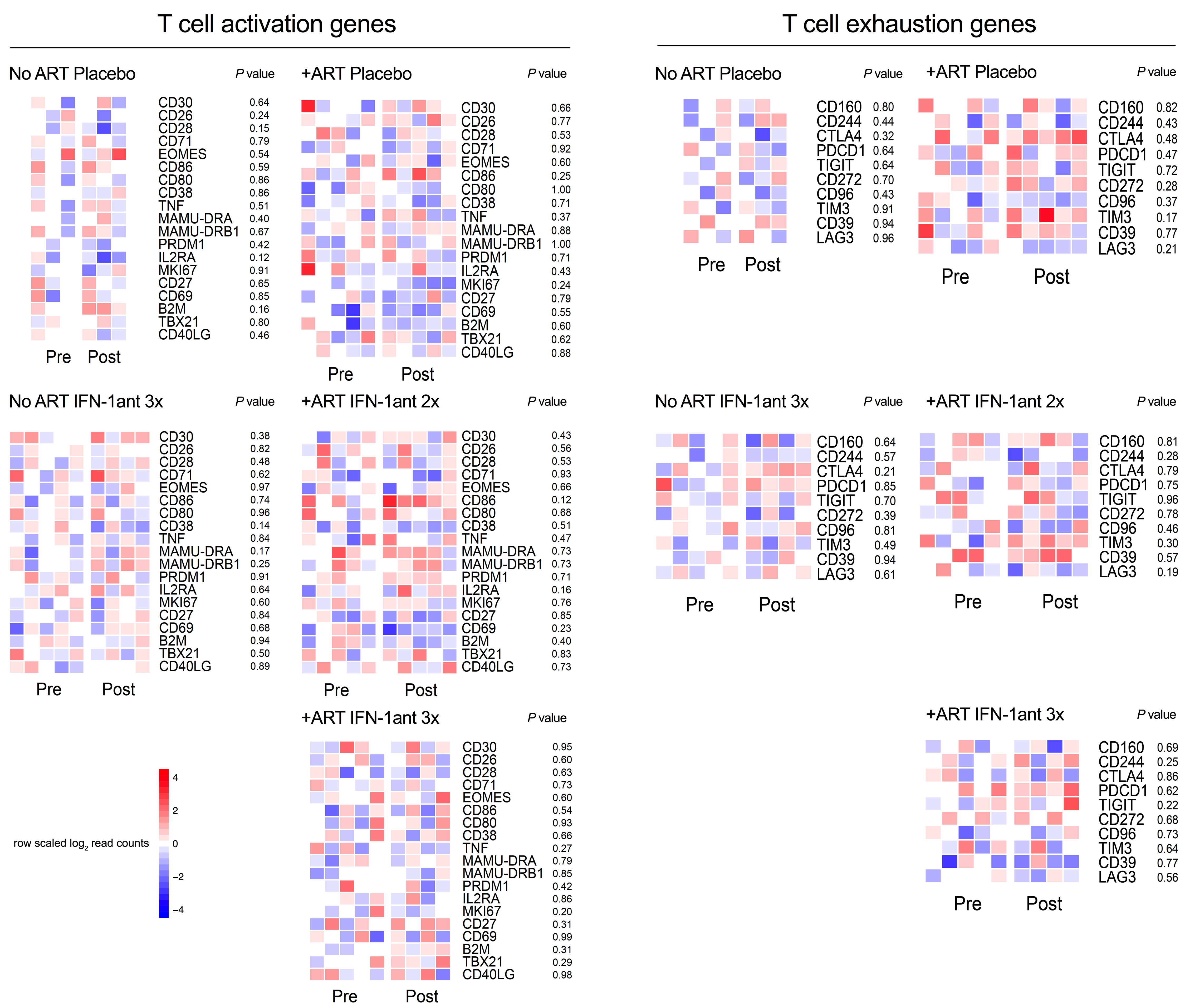

Supplement: S6 Fig — Heatmaps indicate expression of select genes associated with T cell activation, or exhaustion/inhibition at time points prior to (Week 14 post-infection) and following (Week 19 post-infection) IFN-1ant treatment. Heatmap colors represent gene expression levels that were log2 transformed (Variance stabilizing transformation, DESeq2) and normalized to the Week 14 median expression level of each transcript. Statistical significance of differences between Week 14 and Week 19 expression levels for each transcript was determined using negative binomial generalized linear models (DESeq2). (TIF) [file ppat.1007246.s006.tif]
