# Supplementary material for: Type I IFN signaling blockade by a PASylated antagonist during chronic SIV infection suppresses specific inflammatory pathways but does not alter T cell activation or virus replication
Source: PLoS Pathog. 2018 Aug 24;14(8):e1007246. doi: 10.1371/journal.ppat.1007246 (PMC6126880; doi:10.1371/journal.ppat.1007246)
Supplement: S1 Table — (DOCX) [file ppat.1007246.s007.docx]

**S1 Table. CMV plasma virus load before and after administration of PASylated antagonist.**

|  | | CMV copies/ml | | Group proportion CMV^+^ | |
| --- | --- | --- | --- | --- | --- |
|  |  | Pre-  IFN-1ant | Post-  IFN-1ant | Pre-  IFN-1ant | Post-  IFN-1ant |
| +ART  Placebo | A10V012 | 6250 | 0 | 2/5 | 1.5 |
|  | A11E030 | 23352 | 17404 |  |  |
|  | ZJ06 | 0 | 0 |  |  |
|  | ZJ07 | 0 | 0 |  |  |
|  | ZJ53 | 0 | 0 |  |  |
| +ART  IFN-1ant 2x | A9P012 | 0 | 0 | 3/11 | 1/11 |
|  | A9V044 | 0 | 0 |  |  |
|  | A10E060 | 27711 | 12526 |  |  |
|  | A10E109 | 0 | 0 |  |  |
|  | A11E008 | 0 | 0 |  |  |
|  | A11E078 | 0 | 0 |  |  |
|  | BG58 | 6915 | 0 |  |  |
|  | BG73 | 0 | 0 |  |  |
|  | ZH40 | 0 | 0 |  |  |
|  | ZH56 | 5147 | 0 |  |  |
|  | ZJ24 | 0 | 0 |  |  |
| +ART  IFN-1ant 3x | 23212 | 0 | 0 | 1/5 | 0/5 |
|  | 25011 | 0 | 0 |  |  |
|  | 26311 | 0 | 0 |  |  |
|  | 26711 | 0 | 0 |  |  |
|  | 29512 | 2832 | 0 |  |  |
