# Supplementary material for: Type I IFN signaling blockade by a PASylated antagonist during chronic SIV infection suppresses specific inflammatory pathways but does not alter T cell activation or virus replication
Source: PLoS Pathog. 2018 Aug 24;14(8):e1007246. doi: 10.1371/journal.ppat.1007246 (PMC6126880; doi:10.1371/journal.ppat.1007246)
Supplement: S2 Table — (DOCX) [file ppat.1007246.s008.docx]

**S2 Table. Blood chemistry before and after administration of PASylated antagonist**

|  | **+ ART** | | | | | | **No ART** | | | |
| --- | --- | --- | --- | --- | --- | --- | --- | --- | --- | --- |
|  | Before antagonist | | | After antagonist | | | Before antagonist | | After antagonist | |
|  | Placebo | IFN-1ant 2x | IFN-1ant 3x | Placebo | IFN-1ant 2x | IFN-1ant 3x | Placebo | IFN-1ant 3x | Placebo | IFN-1ant 3x |
| Sodium mmol/L | 148  (147-151) | 149  (145-153) | 148.5  (146-158) | 148  (146-150) | 149  (147-152) | 149  (149-150) | 151  (147-153) | 147.5  (140-151) | 151.5  (148-155) | 149  (146-152) |
| Potassium mmol/L | 3.7  (3.1-4.2) | 3.8  (2.9-5.1) | 4.5  (3.3-5.1) | 3.5  (3.0-3.9) | 3.3  (2.8-4.0) | 4.1  (3.8-4.2) | 3.3  (3.0-3.4) | 4.3  (3.0-5.7) | 3.6  (3.4-3.8) | 3.5  (3.2-4.1) |
| Chloride mmol/L | 104  (101-108) | 104  (102-110) | 104.5  (102-111) | 105  (102-109) | 105  (102-110) | 105  (101-109) | 105.5  (105-107) | 103.5  (95-105) | 103.5  (103-106) | 103  (102-108) |
| Calcium mmol/L | 2.6  (2.3-2.7) | 2.5  (2.4-2.7) | 2.6  (2.5-2.7) | 2.4  (2.2-2.7) | 2.3  (2.2-2.6) | 2.5  (2.4-2.6) | 2.3  (2.2-2.5) | 2.6  (2.3-2.6) | 2.3  (2.2-2.5) | 2.5  (2.4-2.9) |
| Magnesium mmol/L | 0.8  (0.7-0.9) | 0.8  (0.7-1.0) | 0.8  (0.8-0.9) | 0.8  (0.6-0.9) | 0.9  (0.8-1.0) | 0.8  (0.8-0.8) | 0.8  (0.7-0.8) | 0.8  (0.7-0.8) | 0.7  (0.7-0.7) | 0.8  (0.7-0.9) |
| Phosphorus mg/dL | 5.1  (4.1-8.0) | 4.8  (3.5-7.4) | 4.2  (2.7-5.4) | 4.8  (0.8-7.4) | 4.8  (1.4-7.5) | 4.3  (3.3-5.2) | 5.5  (4.4-7.8) | 2.7  (1.5-6.2) | 5.7  (4.4-7.5) | 2.7  (2.3-6.5) |
| Glucose mg/dL | 36.0  (28-54) | 32.0  (25-46) | 50.5  (30-69) | 49  (13-83) | 41  (15-72) | 50  (38-61) | 47  (40-53) | 37.5  (11-61) | 56  (46-63) | 54  (28-64) |
| BUN  mg/dL | 14  (10-29) | 13  (9-25) | 13  (10-17) | 13  (8-24) | 16  (10-22) | 13  (9-19) | 16.5  (14-17) | 15.5  (13-25) | 16.5  (15-18) | 15  (10-19) |
| Creatinine mg/dL | 0.9  (0.7-1.2) | 0.9  (0.8-1.3) | 1.1  (0.8-1.4) | 1.0  (0.8-1.1) | 1.0  (0.8-1.5) | 1.0  (0.8-1.4) | 0.7  (0.6-0.9) | 1.0  (0.8-1.3) | 0.7  (0.5-0.8) | 0.8  (0.7-1.2) |
| Uric Acid mg/dL | < 0.2 | < 0.2 | < 0.2 | < 0.2 | < 0.2 | < 0.2 | < 0.2 | < 0.2 | < 0.2 | < 0.2 |
| Albumin  g/dL | 4.6  (3.6-5.1) | 4.3  (4.0-4.8) | 4.6  (4.3-4.9) | 4.5  (4.3-4.9) | 4.6  (4.2-5.0) | 4.4  (4.1-4.6) | 4.1  (3.5-4.8) | 4.4  (4.2-5.2) | 4.2  (3.6-4.5) | 4.4  (3.9-4.6) |
| Total Protein g/dL | 7.0  (6.4-7.3) | 7.0  (6.7-7.9) | 7.3  (7.2-7.5) | 7.0  (6.7-7.4) | 7.4  (7.0-7.7) | 7.1  (6.8-7.3) | 7.7  (7.4-8.3) | 7.1  (6.7-7.6) | 7.6  (7.4-8.0) | 7.2  (5.9-7.7) |
| Cholesterol mg/dL | 143  (122-183) | 148  (114-201) | 148.5  (111-169) | 167  (135-202) | 153  (129-188) | 137  (99-156) | 156  (131-161) | 152  (80-203) | 151.5  (128-160) | 152  (125-164) |
| Triglycerides mg/dL | 57  (36-108) | 68  (32-98) | 58.5  (37-77) | 50  (26-79) | 55  (38-83) | 68  (34-73) | 52.5  (40-68) | 50.5  (42-153) | 47.5  (36-61) | 83  (47-115) |
| Alk. Phos.  U/L | 615  (222-929) | 448  (181-999) | 351  (328-449) | 631  (234-986) | 365  (137-882) | 359  (266-558) | 370.5  (136-672) | 245.5  (92-386) | 463  (215-720) | 336  (126-450) |
| ALT  U/L | 30  (21-120) | 26  (19-49) | 31.5  (20-40) | 30  (19-52) | 23  (18-51) | 25  (18-36) | 21.5  (18-22) | 28.5  (17-70) | 20  (19-22) | 27  (19-40) |
| AST  U/L | 60  (36-89) | 56  (33-70) | 52.5  (34-72) | 47  (37-63) | 49  (35-67) | 51  (41-59) | 40  (36-44) | 49  (42-63) | 39.5  (28-45) | 45  (42-49) |
| Amylase  U/L | 385  (110-521) | 421  (285-569) | 354.5  (335-389) | 337  (285-523) | 393  (251-481) | 382  (294-418) | 334  (304-467) | 371  (269-649) | 334.5  (282-304) | 409  (415-639) |
| CK total  U/L | 380  (137-1974) | 284  (140-738) | 349  (165-565) | 426  (188-1775) | 429  (203-746) | 411  (192-588) | 631  (452-935) | 446.5  (285-575) | 475  (207-685) | 430  (349-585) |
| LD  U/L | 872  (522-1210) | 996  (473-1281) | 1151.5  (781-1688) | 890  (580-1056) | 900  (589-1164) | 1176  (787-1314) | 651.5  (411-1211) | 958  (719-1087) | 677  (426-992) | 763  (677-1051) |

### Values indicate median and range. ALT = Alanine Aminotransferase; AST = Aspartate Aminotransferase; CK = creatine kinase; LD = lactate dehydrogenase
