# Supplementary material for: Type I IFN signaling blockade by a PASylated antagonist during chronic SIV infection suppresses specific inflammatory pathways but does not alter T cell activation or virus replication
Source: PLoS Pathog. 2018 Aug 24;14(8):e1007246. doi: 10.1371/journal.ppat.1007246 (PMC6126880; doi:10.1371/journal.ppat.1007246)
Supplement: S3 Table — (DOCX) [file ppat.1007246.s009.docx]

**S3 Table. T cell Exhaustion and Activation GSEA FDR q-values**

|  | **+ART IFN1-ant2x** | **+ART IFN1-ant3x** | **+ART Placebo** | **No ART IFN1-ant3x** | **No ART Placebo** |
| --- | --- | --- | --- | --- | --- |
|  |  |  |  |  |  |
| **T cell Exhaustion Gene Sets** |  |  |  |  |  |
| upregulated in exhausted cells ^1^ | 0.68 | 0.82 | 0.53 | 0.03 | 0.51 |
| downregulated in exhausted cells ^2^ | 0.28 | 0.82 | 0.56 | 0.46 | 0.51 |
| upregulated in exhausted cells ^3^ | 0.88 | 1 | 0.83 | 0.87 | 0.38 |
| downregulated in exhausted cells ^4^ | 0.9 | 0.63 | 0.89 | 1 | 0.16 |
| upregulated in exhausted cells ^5^ | 0.87 | 0.99 | 0.28 | 0.14 | 0.81 |
| upregulated in exhausted cells ^6^ | 0.63 | 1 | 0.86 | 0.11 | 0.85 |
| **T cell Activation Gene Sets** |  |  |  |  |  |
| Upregulated in activated T cells ^7^ | 0.95 | 0.87 | 0.62 | 0.78 | 0.42 |
| Upregulated in activated T cells ^8^ | 0.26 | 0.24 | 0.32 | 0.9 | 0.42 |
| Upregulated in activated T cells ^9^ | 0.31 | 0.99 | 0.59 | 0.43 | 0.49 |
| T cell receptor signaling molecules ^10^ | 0.43 | 0.19 | 0.15 | 0.46 | 0.2 |
| Upregulated in activated T cells ^11^ | 0.97 | 0.7 | 0.49 | 0.91 | 0.21 |

Values indicate FDR q-values of GSEA analyses comparing weel 14 to week 19 samples using gene sets derived from experiments identifying genes associated with T cell activation or exhaustion

^1^ MSigDB M4540 GSE24081_CONTROLLER_VS_PROGRESSOR_HIV_SPECIFIC_CD8_TCELL_DN

^2^ MSigDB M4539 GSE24081_CONTROLLER_VS_PROGRESSOR_HIV_SPECIFIC_CD8_TCELL_UP

^3^ Top upregulated in exhausted as compared to naïve T cells. (Doering et al., Immunity. 2012 December 14; 37(6): 1130–1144. Doi:10.1016.)

^4^ Top downregulated in exhausted as compared to naïve T cells. (Doering et al., Immunity. 2012 December 14; 37(6): 1130–1144. doi:10.1016.)

^5^ T cell exhaustion genes consistent across tumors, Fig. 5E (Tirosh et al., Science. 2016 April 8; 352(6282): 189–196. doi:10.1126.)

^6^ T cell exhaustion genes variable across tumors, Fig. 5F (Tirosh et al., Science. 2016 April 8; 352(6282): 189–196. doi:10.1126.)

^7^ Genes with expression correlating with CD8ki67 in SIV infected rhesus macaques (Rotger et al., J Clin Invest. 2011 Jun;121(6):2391-400. doi: 10.1172.)

^8^ T cell activation gene modules (M5.1, M7.1, M7.3, M7.4, Li et al., Nat Immunol. 2014 Feb;15(2):195-204. doi: 10.1038.)

^9^ MSigDB M3525 GSE15324_NAIVE_VS_ACTIVATED_CD8_TCELL_DN

^10^ MSigDB M19784 BIOCARTA_TCR_PATHWAY

^11^ MSigDB M3036 GOLDRATH_NAIVE_VS_EFF_CD8_TCELL_DN
